# A Lightweight Beryllium Metal–Organic Framework for Combined Physical and Chemical Hydrogen Storage

**DOI:** 10.1021/acsaem.5c02864

**Published:** 2025-10-31

**Authors:** Giacomo Provinciali, Naomi Anna Consoli, Martino Degli Innocenti, Anna Moliterni, Heryson Tresmann, Rocco Caliandro, Cinzia Giannini, Rolando Pedicini, Giuliano Giambastiani, Giulia Tuci, Moreno Lelli, Andrea Rossin

**Affiliations:** † 428773 Istituto di Chimica dei Composti Organometallici (CNR-ICCOM), Via Madonna del Piano 10, Sesto Fiorentino (Firenze) 50019, Italy; ‡ Centre of Magnetic Resonance (CERM) Università di Firenze, Via Luigi Sacconi 6, Sesto Fiorentino (Firenze) 50019, Italy; § Dipartimento di Chimica “Ugo Schiff”, 9300 Università di Firenze, Via della Lastruccia 3-13, Sesto Fiorentino (Firenze) 50019, Italy; ∥ Consorzio Interuniversitario Risonanze Magnetiche Metallo Proteine (CIRMMP), Via Luigi Sacconi 6, Sesto Fiorentino (Firenze) 50019, Italy; ⊥ 296400 Istituto di Cristallografia (CNR-IC), via Amendola 122/o, Bari 70126, Italy; # 201828 CNR-ITAE, Institute for Advanced Energy Technologies, Via S. Lucia sopra Contesse 5, Messina 98126, Italy; ∇ Consorzio Interuniversitario Nazionale per la Scienza e Tecnologia dei Materiali (INSTM), Via G. Giusti, Firenze 950121, Italy

**Keywords:** metal−organic frameworks (MOFs), beryllium, hydrogen storage, ammonia borane, X-ray powder diffraction (XRPD), solid-state NMR spectroscopy

## Abstract

In this work, we report the synthesis and structural characterization of the beryllium-based metal–organic framework of general formula [Be_4_O­(BDC-NH_2_)_2.5_(OAc)] (**Be_BDC_NH**_**2**_), designed for combined physical and chemical hydrogen storage applications. The material was extensively characterized through a plethora of solid-state techniques (including less conventional ^9^Be NMR-MAS spectroscopy). Structural analysis by X-ray powder diffraction confirmed the formation of a crystalline porous framework of **fcu** topology isostructural to MOF-5 and to MOF-5­(Be), while thermogravimetric studies revealed remarkable thermal stability up to 830 K. Nitrogen adsorption measurements demonstrated a high specific surface area (2264 m^2^/g after removal of residual acetic acid), confirming the accessible porosity of the material. Hydrogen adsorption experiments (physical hydrogen storage) performed at cryogenic temperatures showed fast, fully reversible physisorption with a gravimetric H_2_ density of 8.0 wt % H_2_ (*T* = 77 K, *p*_H_2__ = 80 bar) and a H_2_ isosteric heat of adsorption of 2.7 kJ/mol (at 0.1 wt % H_2_ coverage), consistent with weak, noncovalent interactions between the hydrogen molecules and the framework. To enable chemical hydrogen storage, ammonia borane (NH_3_·BH_3_, AB, 19.6 wt % H) was successfully impregnated into the MOF pores by suspending it on concentrated methanol solutions of AB. Solid-state multinuclear (^11^B, ^15^N) NMR spectroscopy revealed the presence of several boron-containing species, indicating partial chemical transformations of ammonia borane within the framework triggered by the formation of an initial B–H···H–N dihydrogen bonding interaction with the amino dangling group on the MOF linker. ^11^B NMR quantification determined a maximum hydride loading of 2.1 AB molecules per formula unit. To our knowledge, this is the first example of a beryllium MOF able to host either physisorbed molecular hydrogen or chemically bound hydrogen in the form of BN-based lightweight inorganic hydrides, highlighting its potential as a multifunctional material for advanced hydrogen storage strategies.

## Graphical abstract

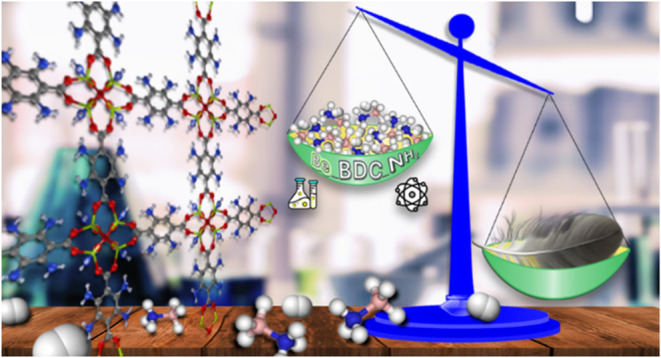

## 1 Introduction

Hydrogen is increasingly recognized as a key element in the transition toward a more sustainable and low-carbon energy future. As a clean and versatile energy carrier, hydrogen can be produced from various renewable and nonrenewable sources and used in a wide range of applications, including fuel cells, industrial processes, and transportation.^1–6^ It offers high gravimetric energy density and can be produced from a variety of renewable sources, such as water electrolysis powered by solar or wind energy. Unlike conventional fossil fuels, the combustion or electrochemical use of hydrogen produces only water as a byproduct, making it an environmentally friendly alternative. However, one of the major challenges associated with the hydrogen economy lies in the efficient and safe hydrogen storage. Due to its low volumetric energy density in the gaseous state, hydrogen must be stored either as molecular H_2_ in a compressed or liquefied form within high-pressure tanks, cryogenic systems and porous solids (physical storage)^7–9^ or chemically bound to lightweight elements (B, N) to form complex metal hydrides (chemical storage).^10–15^ Physical storage involves the compression of hydrogen gas at high pressures (typically 350–700 bar) or liquefaction at cryogenic temperatures (20 K). While mature, these methods face issues related to safety, cost, and energy efficiency. Chemical storage, on the other hand, involves storing hydrogen in the form of chemical compounds that can release hydrogen upon heating. Among the most promising chemical storage materials are lightweight BN-based compounds. In particular, ammonia borane (NH_3_·BH_3_, AB) has a hydrogen content of 19.6 wt % and releases hydrogen in a stepwise thermolysis process. Its relatively low decomposition temperature and high hydrogen density make it attractive. A class of materials widely exploited for either physical (H_2_ physisorption)^16–19^ or chemical (in the form of [AB@MOF] composites)^20^ hydrogen storage is that of Metal–Organic Frameworks (MOFs), crystalline materials composed of metal ions or clusters coordinated to organic ligands, forming extended porous networks.^21–24^ Although less explored compared to the (more conventional) transition metal-based MOFs, beryllium-containing MOFs with their exceptionally low framework densities and high gravimetric surface areas are particularly advantageous for hydrogen storage.^25–27^ The light atomic weight of beryllium contributes to a high gravimetric hydrogen uptake, as the framework mass is minimized relative to the amount of hydrogen adsorbed. With the aim of finding new MOF materials for hydrogen storage applications and taking inspiration from the synthesis of MOF-5­(Be) reported by the group of Mertens in 2010,^26^ we decided to exploit the same methodology to prepare the beryllium MOF containing the 2-aminoterephthalic acid linker (H_2_BDC-NH_2_) of general formula [Be_4_O­(BDC-NH_2_)_2.5_(OAc)] (**Be_BDC_NH**_**2**_). The MOF is isostructural with MOF-5­(Be); it has been fully characterized in the solid state with many complementary techniques and tested for a *combined* physical and chemical hydrogen storage. In physical storage, H_2_ isotherms have been collected on the preactivated sample at (*p*_max_)_H_2__ = 80 bar and *T* = 77/87 K, while in chemical storage the [AB@**Be_BDC_NH**_**2**_] composite has been prepared through impregnation of the pristine MOF with a concentrated AB solution in methanol. To our knowledge, tests of either physical or chemical hydrogen storage within the same material are unprecedented in the literature.

## 2 Experimental Section

### 2.1 Materials and Methods

*Caution: Beryllium compounds are poisonous. All precautions must be taken to avoid skin contact and inhalation of any material containing beryllium. All waste should be treated accordingly and regulations for the disposal of toxic material must be followed. All experiments were carried out in well ventilated hoods* *and wearing FFP2 protective masks*. Beryllium chloride (BeCl_2_), ammonia-borane (NH_3_·BH_3_, AB), 2-aminoterephthalic acid (H_2_BDC-NH_2_) and *N*,*N*-dimethylformamide (DMF) were purchased from Sigma-Aldrich and used as received. Methanol (Aldrich) was distilled on Mg turnings under an N_2_ atmosphere before use and always handled under inert conditions. The starting material beryllium acetate basic [Be_4_O­(OAc)_6_] was prepared according to the synthesis reported by Besson and Hardt et al. in 1954 ^28^ through recrystallization of beryllium hydroxide Be­(OH)_2_ from a hot glacial acetic acid solution. Be­(OH)_2_ was in turn prepared via precipitation from an aqueous solution (deionized water) of BeCl_2_ treated with two equivalents of Na­(OH). The white jelly solid formed was filtered and dried in the oven at 373 K overnight. FT-IR spectra (KBr pellets) were recorded on a PerkinElmer Spectrum BX Series FTIR spectrometer, in the 4000–400 cm^–1^ range, with a 2 cm^–1^ resolution. Thermogravimetric analyses (TG-DTG) were performed under a N_2_ flow (100 mL min^–1^) at a heating rate of 5 K min^–1^ with an EXSTAR Thermo Gravimetric Analyzer Seiko 6200. The latter was coupled with a ThermoStarTM GSD 301T mass spectrometer for mass analysis of the volatile species. The elemental analyses were performed using a Thermo FlashEA 1112 Series CHNS-O elemental analyzer with an accepted tolerance of ±2% on carbon (C), hydrogen (H), nitrogen (N) and sulfur (S). The nature and purity of all the batches employed for the functional characterization were assessed through X-ray powder diffraction (XRPD). XRPD qualitative measurements were carried out in the 2.0–50.0° 2θ region with a Panalytical X’PERT PRO diffractometer equipped with a Ni filter in the diffracted beam, a PIXcel solid state detector and a sealed X-ray tube (Cu Kα, λ = 1.5418 Å). Slits were used on both the incident (Soller slits aperture: 0.25°; divergence slit aperture: 0.5°) and the diffracted (antiscatter slit aperture: 7.5 mm) beam. The generator was operated at 40 kV and 40 mA. Measurements were carried out in reflection mode, using a spinning circular sample holder (flat stage). Hydrogen adsorption measurements were performed using Sievert-type (volumetric) equipment. The gas storage capacity as a weight percentage of adsorbed gas per gram of adsorbent (wt %) was determined from the adsorption isotherms PCT (Pressure–Composition–Temperature). The data shown are graphed in terms of wt % vs. time. Before starting a new series of measurements, each sample was previously activated for 24 h at 423 K under vacuum to remove any fraction of weakly bound solvent within the sample pores.

### 2.2 Multinuclear Solid-State NMR Analysis

1D and 2D solid-state NMR spectra of **Be_BDC_NH**_**2**_ and [AB@**Be_BDC_NH**_**2**_] were acquired on two spectrometers: a Bruker Avance III spectrometer working at 20.0 T (corresponding to 850 MHz for ^1^H, 214 MHz for ^13^C, 86 MHz for ^15^N nuclear Larmor frequencies) operating with a 3.2 mm triple-resonance MAS probe (^1^H/^13^C/^15^N) at 20 kHz of MAS (unless otherwise specified) at a probe temperature of 270 K, and a Bruker NEO spectrometer working at 16.4 T (701 MHz for ^1^H, 176 MHz for ^13^C, 225 MHz for ^11^B, and 99 MHz for ^9^Be nuclear Larmor Frequencies) operating with a 3.2 mm double-resonance MAS probe (^1^H/^11^B) at 20 kHz and at a probe temperature of 270 K (unless otherwise specified). At 16.4 T, ^1^H π/2 pulses were 2.00 μs for **Be_BDC_NH**_**2**_ and 2.40 μs for [AB@**Be_BDC_NH**_**2**_] (unless otherwise specified); ^11^B π/2 pulse was 3.20 μs (low power regime); ^9^Be π/2 pulse was 6.00 μs. In all the {^1^H}-X CP-MAS experiments ^1^H power was linearly ramped from 70% to 100%, while the X heteronuclei were irradiated at constant power with a specific contact time reported in the figure captions.

At 20.0 T, ^1^H π/2 pulse was 2.40 μs for [AB@**Be_BDC_NH**_**2**_] (unless otherwise specified); ^15^N and ^13^C π/2 pulses were 7.40 and 3.50 μs, respectively. In all the {^1^H}-X CP-MAS experiments ^1^H power was linearly ramped from 70% to 100%, while the X heteronuclei were irradiated constant power with a specific contact time indicated in the figure captions. During acquisition of heteronuclei, ^1^H decoupling was performed using SWf-TPPM or SPINAL64. All 2D ^1^H–X, (X = ^11^B, ^13^C, and ^9^Be), HETCOR spectra were acquired applying a FSLG homonuclear {^1^H–^1^H} decoupling in the undirect dimension. Detailed decoupling and CP powers and all the acquisition parameters are listed in the Supporting Information in Tables S1–S7.

### 2.3 Synthesis of **Be_BDC_NH**_**2**_

Beryllium acetate basic [Be_4_O­(OAc)_6_, FW = 406.32, 0.24 g, 0.6 mmol] and 2-aminoterephthalic acid (H_2_BDC-NH_2_, FW = 181.15, 0.33 g, 1.8 mmol, 3 equiv) were dissolved in 90 mL of DMF under vigorous stirring. After 30 min stirring at ambient conditions, the solution was transferred into a 100 mL Teflon-lined stainless-steel autoclave and heated to 423 K for 2 days. After cooling, the microcrystalline yellow powder of **Be_BDC_NH**_**2**_ was collected, washed with EtOH (3 × 10 mL), petroleum ether (3 × 10 mL) and finally dried under a nitrogen stream at room temperature. Yield: 0.25 g {60%, based on the minimal formula [Be_4_O­(BDC-NH_2_)_2.5_(OAc)]·(HOAc)·DMF}. Elemental analysis calcd. (%) for **Be_BDC_NH**_**2**_, C_27_H_26.5_N_3.5_O_16_Be_4_ (MW = 692.07 g/mol): C 46.8, H 3.9, N 7.1. Elemental analysis found (%): C, 46.5; H, 3.9; N, 6.9. IR bands (KBr pellet, cm^–1^): 1623­(vs) [ν­(COO)_OAc_], 1583­(vs) [ν­(COO)_BDC_NH_2__], 1501­(m), 1450­(vs), 1398(s), 1260­(m), 1112­(w), 973­(w), 886­(m), 792­(vs) [γ­(C–H)], 618­(w).

### 2.4 X-ray Powder Diffraction Data Collection and Structure Determination of **Be_BDC_NH**_**2**_

XRPD data for structure determination were collected with the same diffractometer used for the qualitative measurements (Section 2.1) but with a different setup: the divergence slit aperture was set to 0.125°. The XRPD patterns were recorded in the extended 2θ range 1.5–90°, with 2θ increments of 0.02° (total collection time ∼7 h). XRPD data were successfully analyzed by the software *EXPO*,^29^ a package able to carry out the full structure solution process (*i.e*., indexing, space group determination, extraction of the integrated intensities, structure solution and structure refinement by Rietveld method). Additional applied computer programs were: *Mercury* ^30^ for molecular graphics; *publCIF* ^31^ for preparing the published material. The main details on crystal data, data collection and structure refinement are provided in Tables S8 and S9 of the Supporting Information. Crystallographic data of **Be_BDC_NH**_**2**_ have been deposited at the Cambridge Crystallographic Data Centre (CCDC) with CCDC deposition number 2478232 and can be obtained free of charge via www.ccdc.cam.ac.uk/structures.

### 2.5 Synthesis of [AB@**Be_BDC_NH**_**2**_]

**Be_BDC_NH**_**2**_ (0.61 g, 0.88 mmol) was dispersed in 75 mL of an acetone solution of triethylamine (NEt_3_, 1.2 mL, 8.8 mmol, 10 equiv) and heated at 323 K for 15 h under stirring. The yellow solid was then filtered and washed with fresh acetone (3 × 20 mL). After this preliminary washing, the MOF (0.12 g, 0.20 mmol) was suspended in 20 mL of a distilled MeOH ammonia borane (NH_3_·BH_3_, AB, FW = 30.9, 0.032 g, 1.0 mmol, 5 equiv) solution. The suspension was stirred at room temperature for 24 h under nitrogen (at *p* = 10 bar). The resulting yellow solid [AB@**Be_BDC_NH**_**2**_] was collected, washed with fresh MeOH (2 × 10 mL) and finally dried under a nitrogen stream at room temperature. Yield: 0.12 g.

### 2.6 Textural Properties Assessment through N_2_ Adsorption: H_2_ Adsorption Isotherms

The powdered samples (ca. 40 mg) of **Be_BDC_NH**_**2**_ and [AB@**Be_BDC_NH**_**2**_] were activated at *T* = 423 and 298 K respectively under high vacuum (10^–6^ Torr) for 24 h before the measurement. The Brunauer–Emmett–Teller (BET) specific surface area, pore size distribution and pore volume (*V*_tot_, V_micro_) were estimated by volumetric adsorption with an ASAP 2020 Micromeritics instrument, using N_2_ as adsorbate at 77 K. For the BET specific surface area calculation, the 0.01–0.1 *p*/*p*_0_ pressure range of the isotherm was used to fit the data. Within this range, all the Rouquerol consistency criteria^32,33^ are satisfied. The material (micro)­porosity was determined from the N_2_ adsorption isotherm using a NLDFT method (Tarazona approximation) and assuming a cylindrical pore shape (typical of metal oxides). H_2_ adsorption isotherms were collected with a PCT Pro 2000 volumetric gas sorption analyzer (SETARAM Instrumentation). On preactivated **Be_BDC_NH**_**2**_, kinetic studies were performed at three different temperatures (77, 87, and 298 K) through short adsorption/desorption cycles to verify reversibility over time.

## 3 Results and Discussion

### 3.1 Synthesis and Characterization of **Be_BDC_NH**_**2**_

Beryllium is the first member of the alkaline Earth metal family and, unlike other members of the group, forms tetrahedrally coordinated building units with carboxylate oxygen atoms. This unique feature is attributed to its size and the fact that its bonds are covalent in nature, owing to its comparatively high charge/radius ratio.^27,34^ The Be–O coordination bonds are very strong, and this imparts a high thermal stability to the final product. With the hydrogen storage target in mind, the combination of a lightweight metal like beryllium and an organic linker containing a polar group on its skeleton (it is known from the literature that the presence of polar functional groups enhances molecular hydrogen storage by increasing the strength of physisorption interactions through dipole–induced dipole forces)^35,36^ should lead to a MOF material with optimal performance. Thus, we selected the commercially available 2-aminoterephthalic acid to build the MOF-5­(Be)^26^ analogue **Be_BDC_NH**_**2**_. The synthetic protocol employed is the same as that reported for MOF-5­(Be): a simple acetate-aminoterephthalate exchange starting from the preformed acetate cluster [Be_4_O­(OAc)_6_] (Scheme 1). Beryllium acetate basic is reacted with H_2_BDC-NH_2_ in *N*,*N*-dimethylformamide (DMF) solution under solvothermal conditions (autogenous pressure) in Teflon-lined stainless-steel autoclaves to produce the pure product after 2 days at 323 K and final slow cooling.

**Scheme 1:**
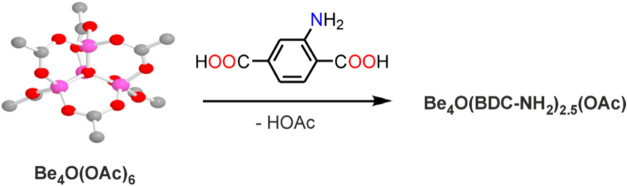
Formation of **Be_BDC_NH**_**2**_ from Beryllium Acetate Basic

The as-obtained white powder was fully characterized in the solid state through a plethora of techniques. The IR spectrum of **Be_BDC_NH**_**2**_ (Figure S1) shows the same ν­(COO)_asym_ band of H_2_BDC-NH_2_ at 1583 cm^–1^, albeit slightly red-shifted with respect to the free linker (1595 cm^–1^), as normally observed for all carboxylic acids upon coordination to a metal ion. Remarkably, another carboxylate ν­(COO)_asym_ band appears in the MOF spectrum at ν = 1623 cm^–1^ that can be ascribed to the acetate ligand (ν = 1637 cm^–1^ in beryllium acetate basic). This is indicative of the presence of acetate ions in the product. Further evidence comes from the thermogravimetric profile (TGA) combined with mass spectrometry (MS) analysis carried out on the exaust (Figure S2a,b, respectively) The MOF decomposition temperature (*T*_dec_ = 830 K) is similar to that of MOF-5­(Be) (∼820 K),^26^ highlighting the remarkable thermal stability of Be-based carboxylate MOFs. The initial weight loss of ca. 19% wt. at *T* = 593 K is ascribed to a combined [DMF (*m*/*z* = 73 a.m.u.) + acetic acid (fragmentation at *m*/*z* = 43 a.m.u.)], followed by complete decomposition at T_dec_. The presence of acetic acid even after thorough washing of the solid in the initial workup protocol possibly stems from a partial *protonation of the basic amino group on the linker* under acidic conditions, producing the ammonium acetate salt *in situ* during the MOF synthesis. This hypothesis is also confirmed by the solid-state ^15^N NMR study (*vide infra*). Another proof of evidence comes from the comparison of the BET surface area of **Be_BDC_NH**_**2**_ before and after washing with a triethylamine (NEt_3_) acetone solution kept at *T* = 323 K for 12 h. The MOF basic treatment that removes HOAc in the pores significantly increases its surface area, passing from 1735 to 2264 m^2^/g (Figure 6). The mass fragmentation peak at *m*/*z* = 43 a.m.u. of acetic acid is also observed at *T*_dec_; this may reveal that a few acetate ions are still coordinated to the [Be_4_O]^6+^ nodes even after the linker exchange reaction. Further proof of evidence comes from the ^9^Be NMR-MAS spectra collected on **Be_BDC_NH**_**2**_ (*vide infra*), where two magnetically distinct Be^2+^ ions are found. From all these considerations (and taking the elemental analysis data into account as well), we propose the minimal formula {[Be_4_O­(BDC-NH_2_)_2.5_(OAc)]·(HOAc)·DMF} for the as-synthesized material. To confirm this formula, we decided to analyze through quantitative solution ^1^H NMR a digested sample (see Supporting Information for details) prepared in the same way, washed and activated to remove as much as possible the clathrate DMF solvent and acetate ions. This NMR analysis revealed the presence of residual acetate in accordance with the minimal formula [Be_4_O­(BDC-NH_2_)_2.76_(OAc)_0.48_], that is close to that found through elemental analysis. This also indicates that the amount of coordinated acetate is batch-dependent. The quantification of acetate through an indirect estimation based on ^9^Be NMR-MAS from the fit of the quadrupolar line shape is not accurate enough to provide a reliable information about the actual stoichiometry. The empty MOF has also been characterized through multinuclear (^1^H, ^13^C, ^9^Be) solid-state NMR spectroscopy. For the 2-aminoterephthalic acid atomic assignment, solution spectra in DMSO-*d*_6_ were also acquired, and the proton and carbon resonances were unambiguously assigned by combining 1D ^1^H, 2D ^1^H–^13^C HSQC, 2D ^1^H–^13^C HMBC, 2D ^1^H–^1^H COSY experiments. The data are reported in the Supporting Information. By comparing solution and solid-state spectra, we fully assigned the ^1^H and ^13^C resonances in the NMR-MAS spectra (Section 3.4–Figure 9). Other signals are observable in the ^13^C NMR-MAS spectrum, and they were assigned to solvents like MeOH, EtOH and DMF. The first was used for the loading with AB (and indeed it is observed only in [AB@**Be_BDC_NH**_**2**_]), while the others come from the washing procedure used in the workup. The thermal activation that is performed on **Be_BDC_NH**_**2**_ before the AB loading step successfully removes the EtOH and DMF present in the as-synthesized MOF batch. As for the protonation state of the −NH_2_ group, the ^1^H NMR spectrum of H_2_BDC-NH_2_ in DMSO-*d*_6_ solution shows a broad singlet at δ_H_ = 8.65 ppm, consistent with the chemical shift of protonated aniline (reported in literature at δ_H_ = 8.66 ppm,^37^ whereas the neutral form appears at δ_H_ = 5.01 ppm^38^). However, the assessment of the real chemical nature of the amino group from the 2D ^1^H–^13^C FSLG HETCOR spectra is challenging because of the signal broadness, dominated by residual homonuclear ^1^H–^1^H dipolar coupling. Acetate/acetic acid species are still present within **Be_BDC_NH**_**2**_ after the ligand exchange synthetic procedure. Acetic acid is released as byproduct from the beryllium acetate basic starting material and it can be trapped in the MOF pores, while some acetate ions can still be present on the metal nodes because the exchange is not complete. The observed aliphatic signals around δ_C_ = 20 ppm (in the carbon dimension) are consistent with such species and are visible also in the ^1^H–^13^C FSLG HETCOR spectrum (Section 3.4–Figure 10), with a δ_H_ chemical shift consistent with the methyl protons of AcOH/AcO^–^. The expected cross-peak between the acetate carbonyl and its methyl group is visible in the ^1^H–^13^C FSLG HETCOR spectrum with very long contact time only (5000 μs, Figure S7), because of the rotational dynamics of the methyl group that reduces the dipolar interactions making the polarization transfer from the methyl protons and the carbonyl ^13^C weak. To have a complementary point of view, the ^9^Be NMR-MAS spectra were also collected. Figures 1 and 2 show the 1D ^9^Be Hahn Echo MAS spectrum and two 2D ^1^H–^9^Be FSLG HETCOR spectra of **Be_BDC_NH**_**2**_, respectively. Since ^9^Be is a quadrupolar nucleus (*I* = 3/2), the observed line width is broadened by the second order quadrupolar interaction that reduces the signal resolution. Conversely, the analysis of the quadrupolar second order line shape and related quadrupolar coupling constant (*C*_Q_) gives information about the geometrical coordination around the metal ion (Figure 3 and Table 1). Evidence for the presence of at least two distinct ^9^Be species within the MOF was obtained from the long contact time 2D ^1^H–^9^Be FSLG HETCOR experiment (Figure 2a), which in turn guided the quadrupolar fitting of the 1D ^9^Be Hahn Echo MAS spectrum. The extracted *C*_Q_ values suggest the presence of two tetrahedral ^9^Be species:^39^ one with higher symmetry at δ_Be_ = 4.45 ppm (lower *C*_Q_ value), assigned to the [Be_4_] node where all the acetate ligands are replaced by (BDC-NH_2_)^2–^ units and another one at δ_Be_ = 4.20 ppm with lower symmetry (higher *C*_Q_ value), where some coordination positions are occupied by residual acetates (lattice defects). This hypothesis is further supported by the 2D ^1^H–^9^Be FSLG HETCOR spectra, particularly when acquired with a short contact time (50 μs, Figure 2b), revealing a series of unshielded protons in close proximity to the beryllium ions. The ^1^H resonance at δ_H_ ≈ 8.5 ppm could be compatible with the aldehydic proton of DMF. The cross-peak at δ_H_ = 5.1 ppm would be consistent with magnetization transfer from adsorbed water molecules.

**Figure 1:**
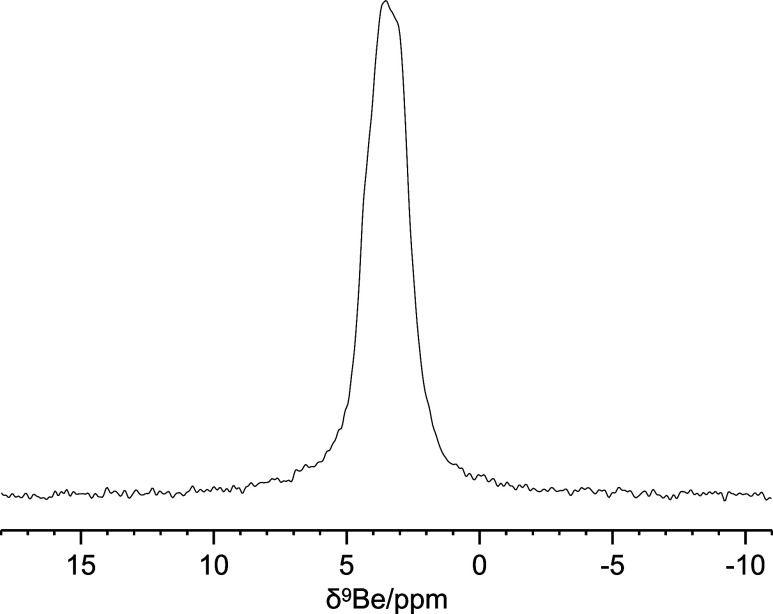
1D ^9^Be Hahn-Echo MAS spectrum of **Be_BDC_NH**_**2**_.

**Figure 2:**
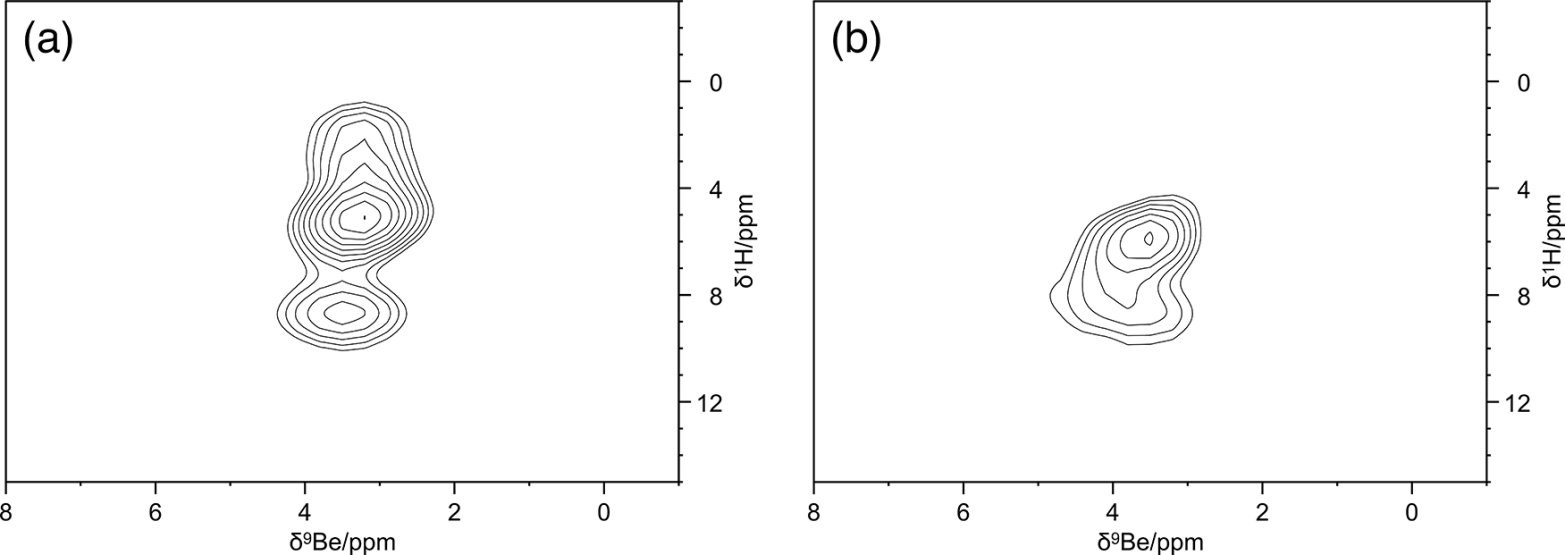
2D ^1^H–^9^Be FSLG HETCOR spectra of **Be_BDC_NH**_**2**_, with a contact time of (a) 1500 μs (long contact time) and (b) 50 μs (short contact time).

**Figure 3:**
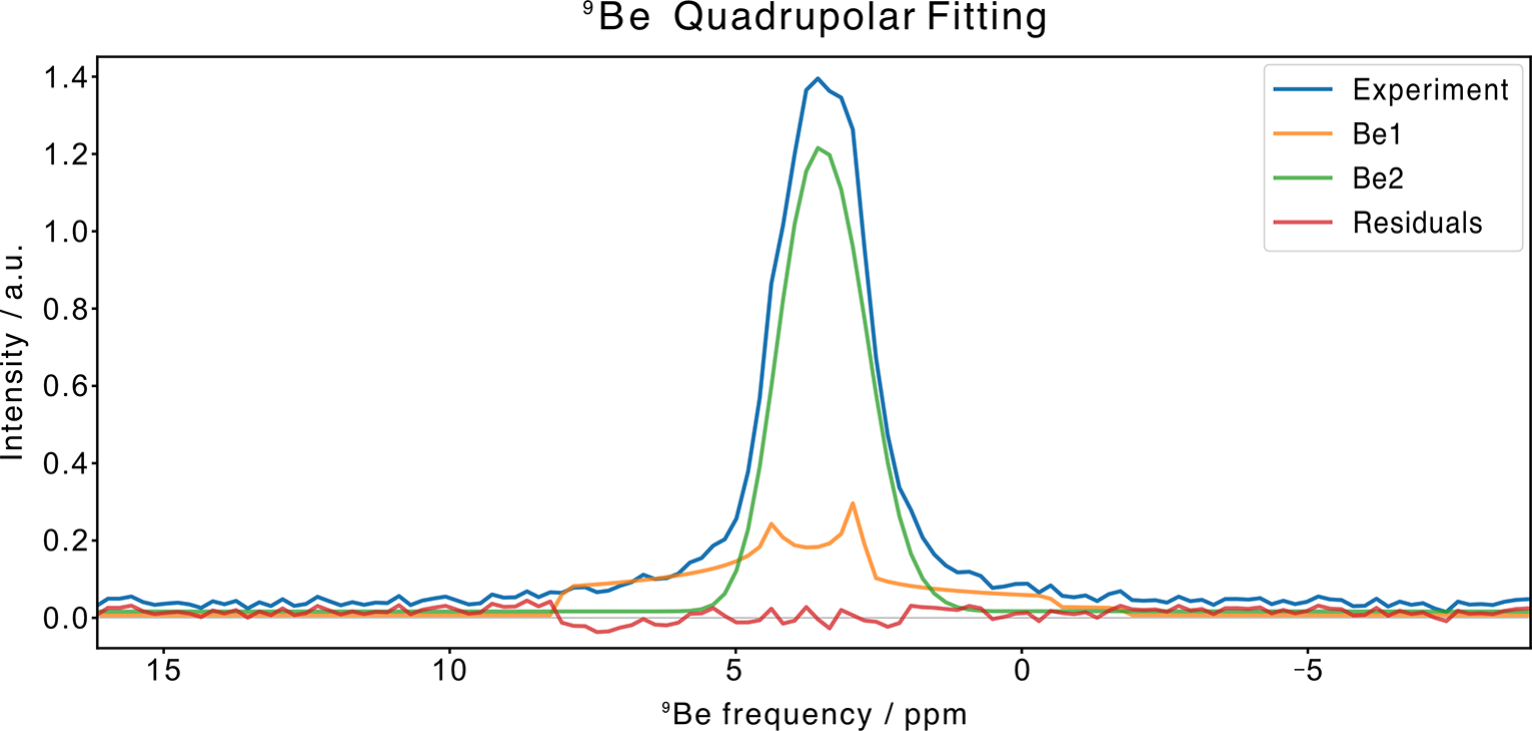
Quadrupolar fitting of 1D ^9^Be Hahn-Echo MAS spectrum of **Be_BDC_NH**_**2**_ performed with the software MrSimulator^40^ under the hypothesis that two magnetically different beryllium species are present within the material.

**Table 1:** ^9^Be Isotropic Chemical Shift (^iso^δ_Be_), Nuclear Electric Quadrupolar Coupling Constant (*C*_Q_) and Asymmetry Parameter (η_Q_) Values Extrapolated from the Fitting of 1D ^9^Be Hahn-Echo MAS Spectrum of **Be_BDC_NH**_**2**_, under the Hypothesis That Two Magnetically Different Beryllium Species are Present within the Material

	^iso^δ_Be_ (ppm)	*C*_Q_ (MHz)	η_Q_	Gaussian FWHM (Hz)
**Be1**	8.20 ± 0.04	1.195 ± 0.005	0.71 ± 0.01	0 ± 1883
**Be2**	4.45 ± 0.02	0.628 ± 0.007	0.02 ± 0.05	120 ± 5

### 3.2 Crystal Structure Determination from X-ray Powder Diffraction Data

All the steps of the *ab initio* structure solution process (*i.e*., indexing, space group determination, extraction of the integrated intensities, structure solution and final refinement using the Rietveld method^41^) were successfully carried out by *EXPO*.^29^ **Be_BDC_NH**_**2**_ crystallizes in cubic crystal system (centrosymmetric space group *Fm*3̅*m*), showing the same **fcu** topology and a very similar crystal structure to that of MOF-5­(Be).^26^ Tables S8 and S9 provide the main crystallographic data and the refined fractional atomic coordinates and isotropic displacement parameters, bond distances and angles. The asymmetric unit of **Be_BDC_NH**_**2**_ consists of seven non-H atoms (Figure 4a); the asymmetric unit and some neighboring symmetry equivalent atoms are visualized in Figure 4b, showing the main building units of the MOF framework, i.e., the [BeO_4_] tetrahedral coordination geometry in the metal node and the linker molecule with a disordered amino group on four C positions of the phenyl ring; a view along the *a* axis of the porous crystal packing is shown in Figure 5a, while the metal node is reported in Figure 5b. The final Rietveld refinement outcome for **Be_BDC_NH**_**2**_ is visualized in Figure S3; the corresponding *R*_p_ and *R*_wp_ agreement factors^42^ are 6.0% and 10.7%, respectively. Details on each step of the structure determination process are summarized in the Supporting Information.

**Figure 4:**
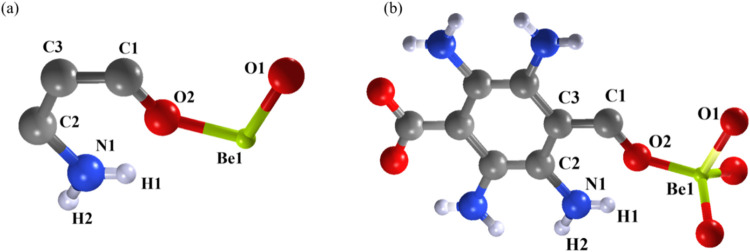
View of the asymmetric unit of **Be_BDC_NH**_**2**_ (a) together with its local environment (b), *i.e*., not labeled neighboring symmetry-equivalent atoms, showing the linker molecule, the disordered –NH_2_ group and the tetrahedral coordination geometry of the metal center. Atom color code: C, gray; H, white; N, blue; O, red; Be, lemon green.

**Figure 5:**
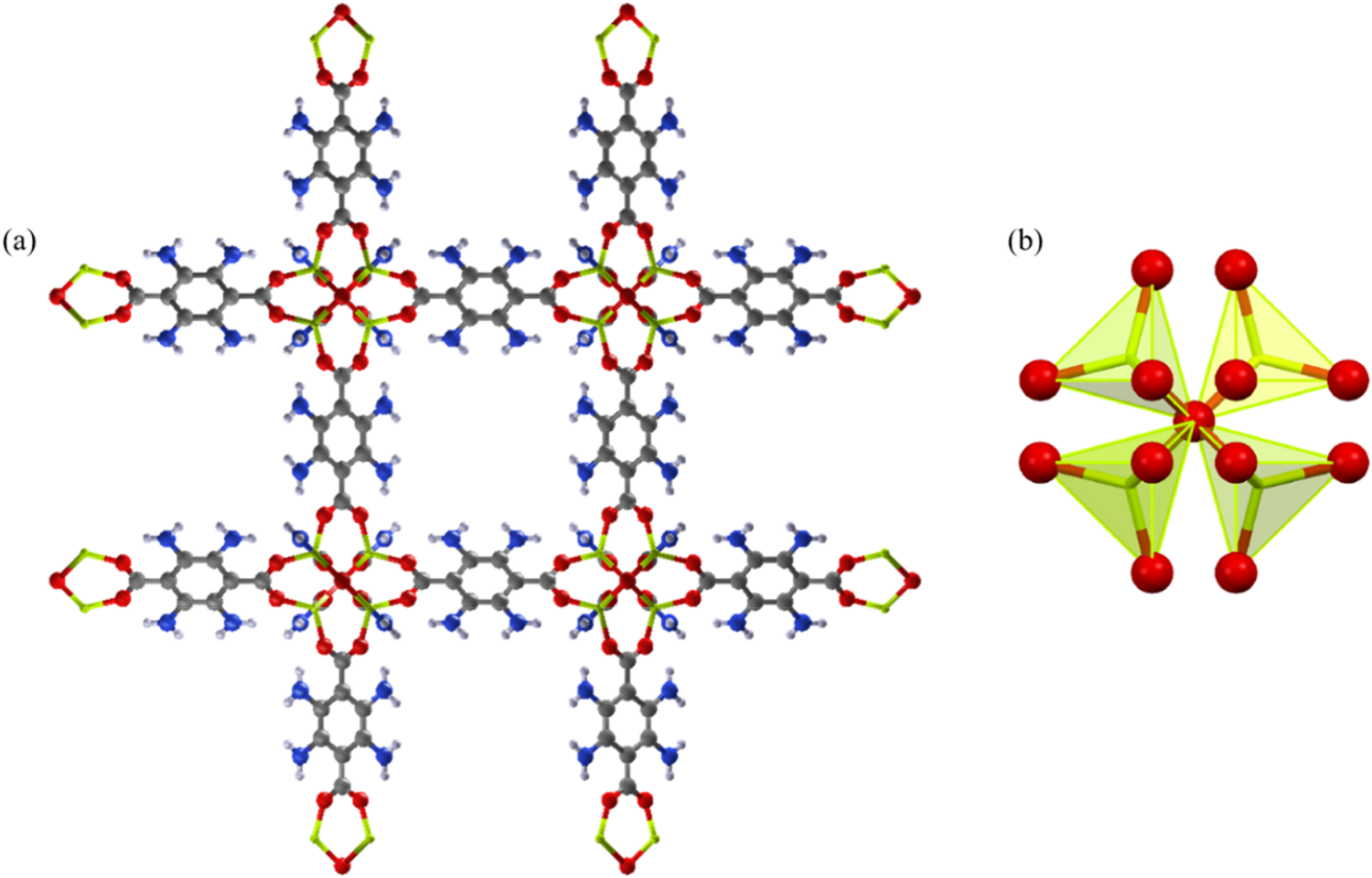
Representation of the crystal structure of **Be_BDC_NH**_**2**_. (a) A view of the 3D open framework visualized along the *a* crystallographic axis. (b) a view of the node (in polyhedral representation). Element color code: C, gray; H, light gray; N, blue; O, red; Be, lemon green.

### 3.3 Textural Properties Assessment: H_2_ Adsorption Isotherms: Physical Hydrogen Storage

The porosity of **Be_BDC_NH**_**2**_ was analyzed through N_2_ volumetric adsorption at 77 K on a preactivated powdered sample (Figure 6a). The isotherm shape is of Type IV, typical of micro- mesoporous materials. The BET specific surface area after basic washing followed by thermal activation at mild temperature equals 2264 m^2^/g; it is lower than that of MOF-5­(Be) (3289 m^2^/g)^26^ or MOF-5 (3235 m^2^/g)^26,43^ as expected when the terephtalate ligand (BDC^2–^) is replaced by the more sterically encumbered BDC-NH_2_^2–^ with dangling amino groups in the pores that reduce the accessible surface area. The mesopore fraction is small, as the micropore volume estimated by the *t*-plot analysis equals 0.74 cm^3^/g (83%) over a total pore volume of 0.89 cm^3^/g. The few mesopores may originate from a partial lattice collapse upon activation. The micropore size distribution evaluated through NLDFT methods has disclosed the presence of micropores in the 14–17 Å size range (Figure 6b).

**Figure 6:**
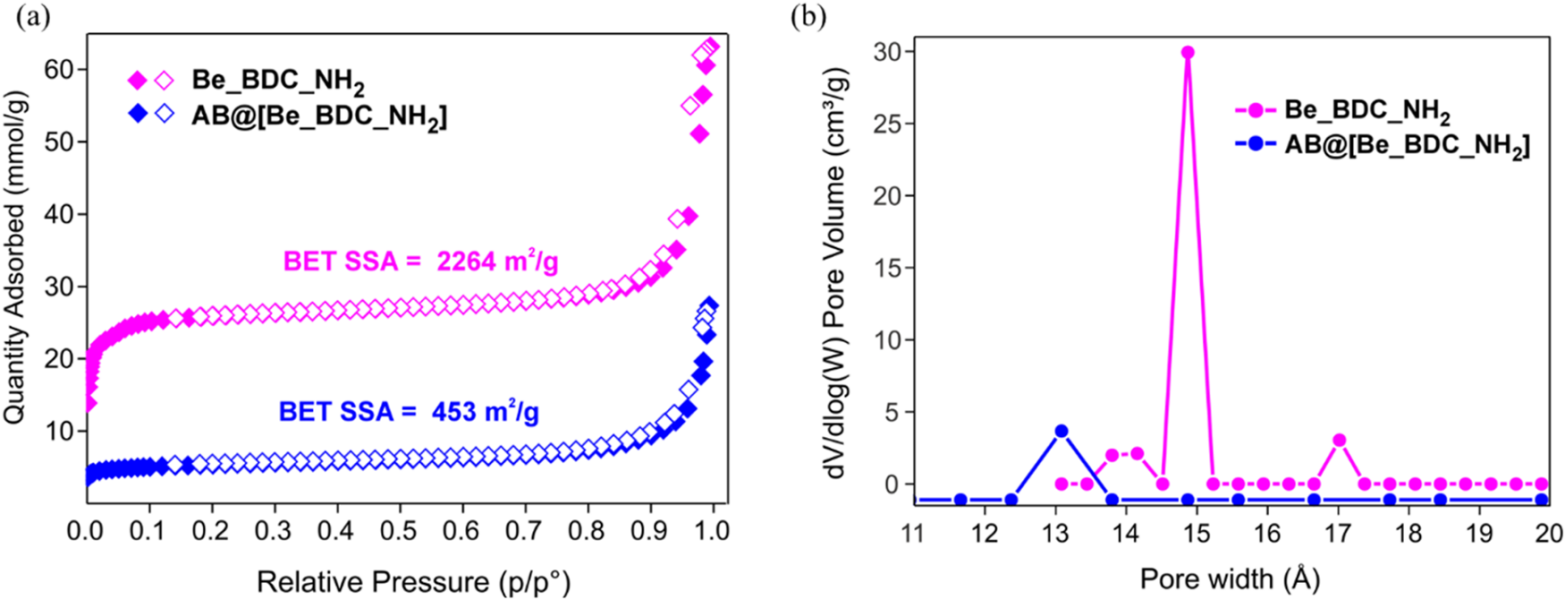
(a) N_2_ isotherms measured at *T* = 77 K on thermally activated **Be_BDC_NH**_**2**_ and AB@[**Be_BDC_NH**_**2**_]. Empty symbols denote the desorption branch. (b) Micropore size distributions (NLDFT method–Tarazona approximation, cylindrical pore shape).

As for hydrogen adsorption, the kinetic measurements carried out on the preactivated MOF at *T* = 77 K (Figure 7a) show the trend at different pressures. In microporous materials with a high surface area, the kinetics are usually very fast to reach saturation. The first step at *p*_H_2__ = 5 bar produces no effect, while in the 5 < *p*_H_2__ < 20 bar range typical hydrogen physisorption can be observed. The processes are sequential; therefore, the total H_2_ storage capacity at *p*_H_2__ = 20 bar and *T* = 77 K (≈0.6 wt % H_2_) is the sum of all steps. The amount adsorbed reaches the remarkable value of ≈8.0 wt % H_2_ at *p*_H_2__ = 80 bar (Figure 7b). This value is comparable to that found for Be-BTB with open metal sites (BTB^3–^ = 1,3,5-benzenetribenzoate, BET area = 4030 m^2^/g) under higher pressure conditions (9.2 wt % H_2_ at *p*_H2_ = 100 bar),^27^ given that the amount of H_2_ adsorbed is proportional to the surface area and the presence of open metal sites (as in Be-BTB) significantly improves H_2_ uptake in MOFs. The desorption process (again featured by a very fast kinetics) shows the same amount of hydrogen adsorbed in the adsorption steps, demonstrating good reversibility. Hydrogen adsorption/desorption cycles performed at *p*_H_2__ = 20 bar and *T* = 77 K (Figure 8) confirmed this behavior. On average, the cycles appear to be reversible without any noticeable degradation in performance.

**Figure 7:**
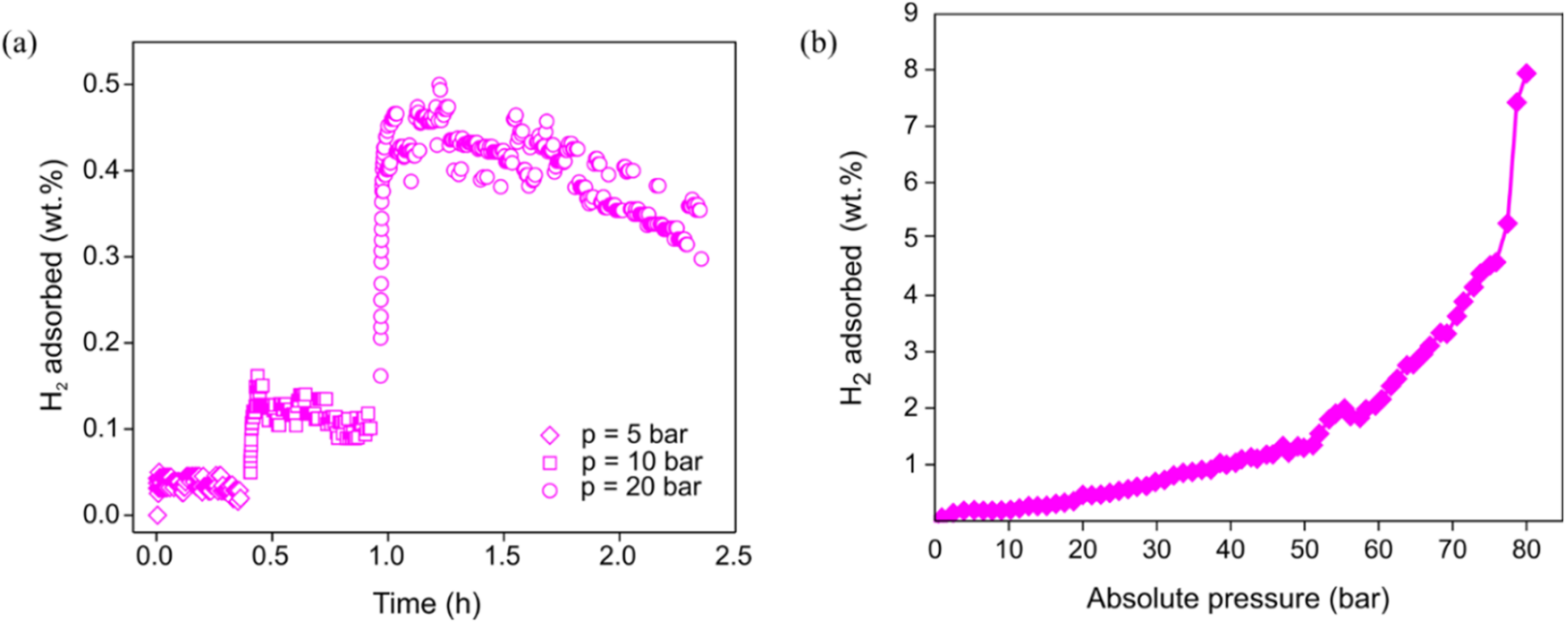
(a) H_2_ adsorption kinetics on **Be_BDC_NH**_**2**_ at *T* = 77 K. (b) H_2_ adsorption isotherm of **Be_BDC_NH**_**2**_ at *T* = 77 K, *p*_H_2__ = 80 bar.

**Figure 8:**
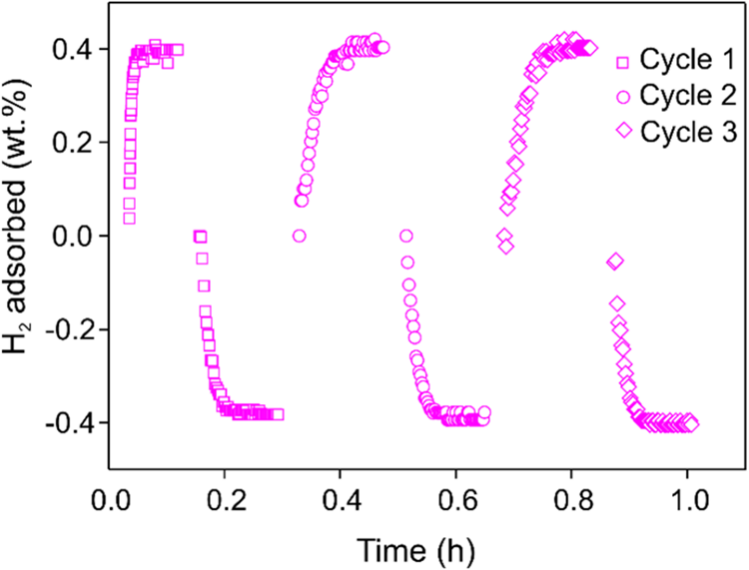
Sequential H_2_ adsorption/desorption cycles carried out on **Be_BDC_NH**_**2**_ at *p*_H_2__ = 20 bar, *T* = 77 K.

The pattern of adsorption/desorption over time of **Be_BDC_NH**_**2**_ does not change when increasing the reaction temperature of 10 K. The cycles performed at *p*_H_2__ = 10 bar and *T* = 87 K (Figure S4) are almost superimposable with the cycles at *T* = 77 K, and they are still completely reversible. Considering the two analysis temperatures of 77 and 87 K, it is possible to calculate the enthalpy (Δ*H*) and entropy (Δ*S*) variations during H_2_ physisorption on **Be_BDC_NH**_**2**_ using the Van’t Hoff equation

1 ln(p)=ΔH/RT−ΔS/R

where *R* = 8.31 J/(mol K) (gas constant). From the corresponding fitting line of the ln­(*p*) vs 1/*T* plot (at the constant coverage of 0.1 wt % H_2_), the ratios Δ*H*/*R* and Δ*S*/*R* were calculated from its slope and intercept, respectively. The enthalpy and entropy variation of the process are −2.7 kJ/mol and −0.05 J/(mol·K), respectively. As typically found for H_2_ adsorbed in MOFs,^44^ the adsorption process is spontaneous and slightly exothermic, albeit the H_2_-MOF interaction is weak. The entropy variation is practically zero, and Δ*G* = Δ*H* – *T*Δ*S* ≈ ΔH. The adsorption enthalpy found falls in the ordinary range found for many MOFs in the literature,^45^ including that found for Be-BTB (−5.5 kJ/mol).^27^ The smaller value of **Be_BDC_NH**_**2**_ may be due to its larger pore size (15–17 Å) if compared with that found in Be-BTB (7 Å). Indeed, calculations proved that the 7 Å diameter is found to be ideal for achieving a high storage density.^46^

### 3.4 Ammonia Borane Loading (Chemical Hydrogen Storage): Synthesis and Characterization of [AB@**Be_BDC_NH**_**2**_]

As already specified in the Introduction, MOFs can serve as hosts to confine ammonia borane molecules to form [AB@MOF] composite materials, enabling better control over AB dehydrogenation kinetics and thermal stability. Furthermore, MOFs can be engineered to include catalytic sites that facilitate the hydrogen release process, potentially lowering the required temperatures and improving the reversibility of the system. This synergistic approach aims to overcome the intrinsic limitations of ammonia borane and move toward more efficient and controllable hydrogen storage technologies. Following this idea, we loaded AB into **Be_BDC_NH**_**2**_ using the conventional impregnation approach suspending the MOF in an AB solution in methanol under 10 bar pressure, to prompt the hydride diffusion through the MOF pores. This synthetic methodology was successfully employed by us in a previous work carried out with zirconium MOFs.^47^ The overall crystal structure and topology of the [AB@MOF] composite is kept unaltered, as inferred from a comparison of the XRPD patterns recorded before and after loading (Figure S5). A slight peak shift to lower 2θ angles is observed, due to an increased cell size when AB is present in the pores. The as obtained [AB@**Be_BDC_NH**_**2**_] composite shows a BET surface area lower than that of pristine **Be_BDC_NH**_**2**_ (453 vs 2264 m^2^/g, Figure 6a), confirming the presence of the hydride within the pores. Additional proof of evidence comes from the pore size distribution of the two samples, where the total pore volume (0.18 cm^3^/g) and the micropore volume at *w* = 13–15 Å are significantly reduced after reaction with AB (Figure 6b). The adduct has also been analyzed through multinuclear (^1^H, ^13^C, ^15^N, ^11^B) solid-state NMR spectroscopy. Figure 9 shows the comparison between the ^13^C Cross-Polarization spectra of **Be_BDC_NH**_**2**_ and [AB@**Be_BDC_NH**_**2**_] with two different contact times. At short contact time {50 μs for **Be_BDC_NH**_**2**_ and 100 μs for [AB@**Be_BDC_NH**_**2**_]}, the carbon atoms bound to protons are mainly observable in the spectrum, whereas at long contact time (1000 μs for both samples) the signals of carbonyl and quaternary carbon atoms are also visible. Therefore, the comparison of the peak intensities from short to long contact times allows for an easy identification of the carbon atoms directly bound to protons (Figure 10).

**Figure 9:**
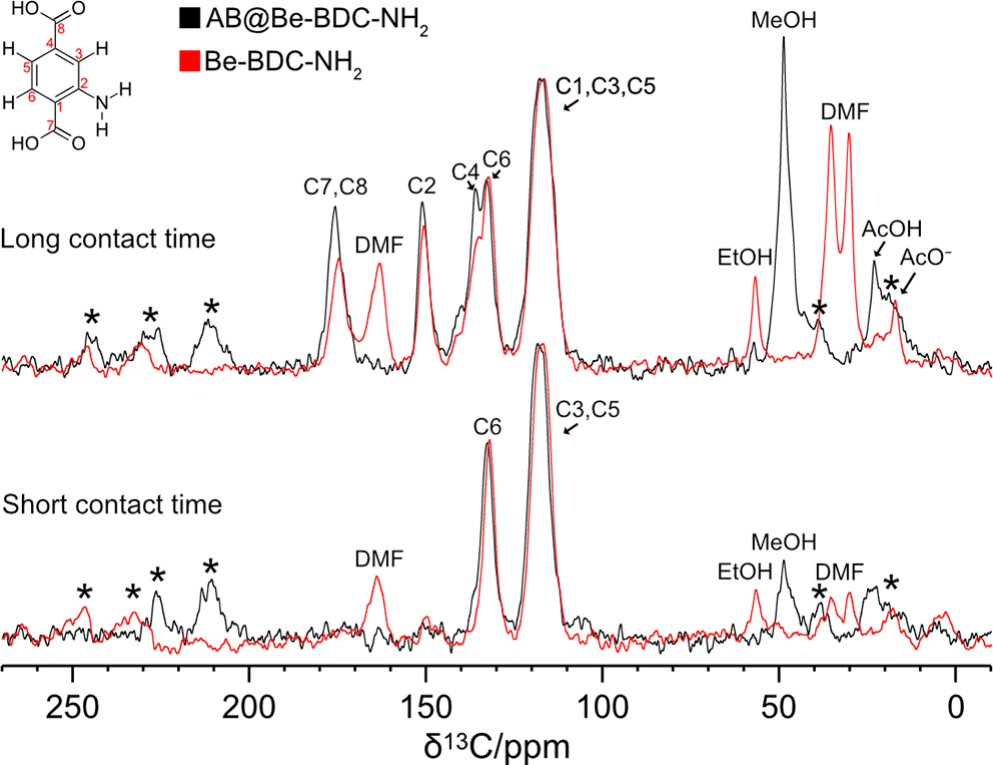
Comparison of 1D {^1^H}-^13^C CP MAS spectra of **Be_BDC_NH**_**2**_ (red) and [AB@**Be_BDC_NH**_**2**_] (black), acquired with two different contact times: 1000 μs (“long” contact time); 50 and 100 μs (“short” contact time). Asterisks denoted spinning sidebands. The spectra are normalized by setting the δ_C_ = 118 ppm peak at the same height.

**Figure 10:**
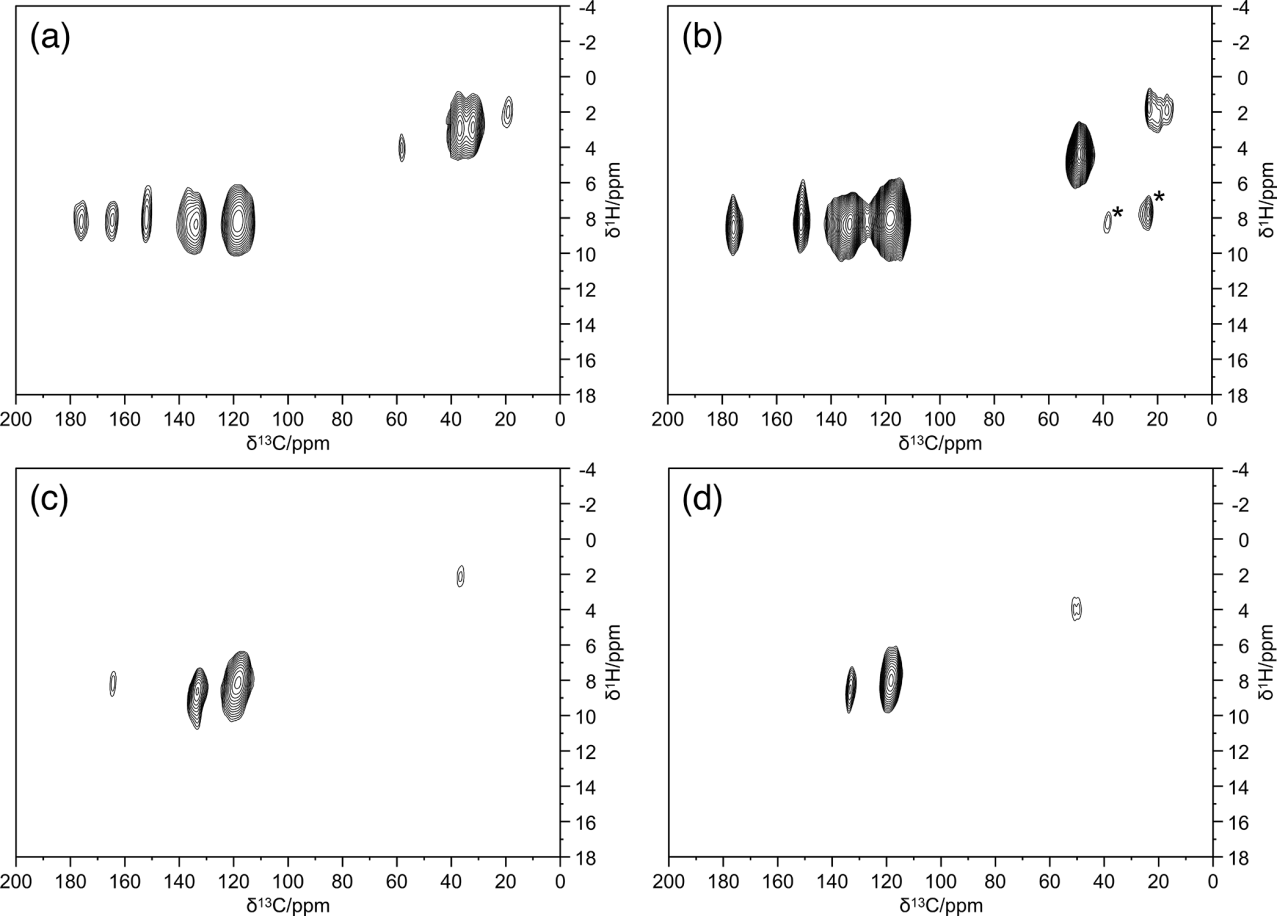
2D ^1^H–^13^C FSLG HETCOR spectra of **Be_BDC_NH**_**2**_ (a, c) and [AB@**Be_BDC_NH**_**2**_] (b, d), acquired with two different contact times: 1000 μs (“long” contact time; a, b); 50 and 100 μs (“short” contact time; c, d). Asterisks denoted spinning sidebands.

On [AB@**Be_BDC_NH**_**2**_], 1D ^11^B Hahn-echo MAS spectrum with ^1^H decoupling (Figure 11) and two 2D ^1^H–^11^B FSLG HETCOR spectra (Figure 12) were collected. The Hahn-echo scheme is particularly useful in solid-state ^11^B NMR to suppress the signal from the probe-head background in the spectrometer (made of boron nitride). Four different main species can be found on the ^11^B spectrum at δ_B_ ≈ 17 ppm (B1), δ_B_ ≈ 2 ppm (B2), δ_B_ ≈ −10 ppm (B3), δ_B_ ≈ −18 ppm (B4) together with a minor peak at δ_B_ ≈ −24 ppm. To get insights into the coordinative geometries of the four major boron species B1–B4, a quadrupolar fitting was performed (Figure 13 and Table 2) through the MrSimulator software developed by Prof. G. Grandinetti of the Ohio University.^40^ Boron is a peculiar case where the isotropic chemical shift together with the quadrupolar coupling constant (*C*_Q_) characterize its coordination geometry. Generally, trigonal boron species (coordination number 3) exhibit isotropic chemical shifts greater than 10 ppm and C_Q_ values higher than 2.5 MHz.^48^ Based on this, we can state that species B1 is trigonal (the ^iso^δ_11B_, C_Q_, η_Q_ found are very similar to those of boric acid),^49,50^ while species B2, B3, and B4 are tetrahedral (coordination number 4). The peak at δ_B_ = −24 ppm can be assigned to unreacted ammonia borane,^47^ possibly interacting via dihydrogen bonding with the amino group of the BDC-NH_2_ linker. The species with isotropic chemical shifts higher than that of B4 could correspond to boron species in which hydrides are progressively replaced by heteroatoms, similarly to what is observed in the ^11^B solution spectra during the test of AB stability in CD_3_OD (Figure S6). In these solution spectra it was also confirmed that the increasing chemical shift correlates with the decrease of the signal multiplicity: from the quartet (*J*_H/B_ = 93 Hz) centered at δ_B_ = −23.3 ppm (AB), to a triplet (*J*_H/B_ = 97 Hz) centered at δ_B_ = −11.7 ppm, a doublet (*J*_H/B_ = 116 Hz) centered at δ_B_ = −5.2 ppm, a weak doublet (*J*_H/B_ = 126 Hz) centered at δ_B_ = 0.3 ppm and finally to broader signals at δ_B_ = 13.9 ppm and δ_B_ = 18.4 ppm. The 2D ^1^H–^11^B FSLG HETCOR spectra (Figure 12) support this hypothesis, showing that B2, B3, and B4 correlate with protons with chemical shifts falling in the typical range of hydride species.^47^ B4 is the only one that does not exhibit correlations with unshielded protons (e.g., OH), further supporting the hypothesis that it closely resembles AB. Thus, we can assign this peak to the product of H_2_ elimination after the initial N–H···H–B dihydrogen bonding interaction between AB and a protonated amino group on the (BDC-NH_3_)^+^ linker, generating the N–B–N short chain (BDC-NH_2_–BH_2_–NH_3_)^+^. The B3 species could be assigned to [BDC-NH_2_–BH_2_(OH)]^+^ while B2 could be [BDC-NH_2_–BH­(NH_3_)­(OH)]^+^. Finally, B1 is most likely fully oxygenated trigonal boron [boric acid, B­(OH)_3_]. To confirm the nature of boronic species bound to the BDC-NH_2_ linker, we acquired a ^1^H–^13^C FSLG HETCOR spectrum with very long contact time of 5000 μs to promote the polarization transfer from ^1^H spins close but not directly connected to carbons. In the spectrum reported in Figure S7 acquired on [AB@**Be_BDC_NH**_**2**_], we can observe the correlations of the BDC-NH_2_ carbons with protons at δ_H_ ≈ 5 ppm from the ammonia part of AB, confirming the proximity of these fragments to the linker. Scheme 2 reports the proposed interaction mechanism between AB and the MOF linker, with the chemical assigment of the various B1-B4 species.

**Figure 11:**
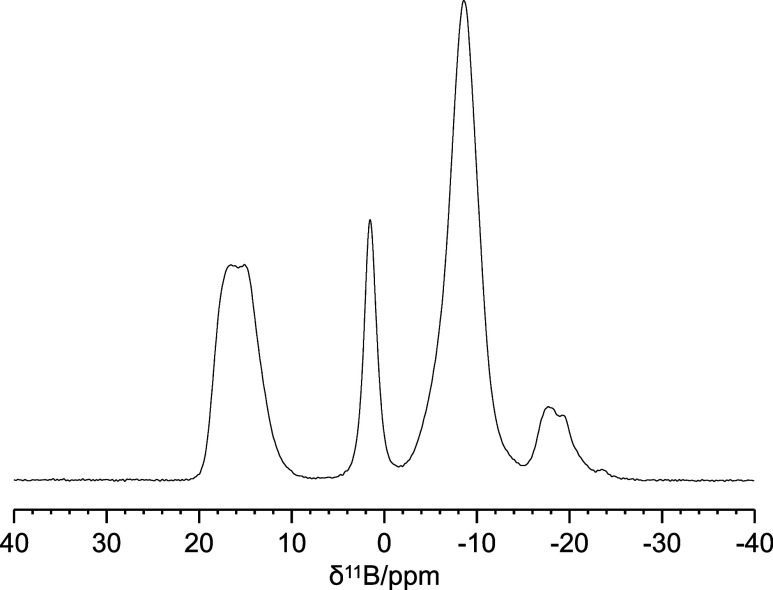
1D ^11^B Hahn-Echo MAS spectrum of [AB@**Be_BDC_NH**_**2**_].

**Figure 12:**
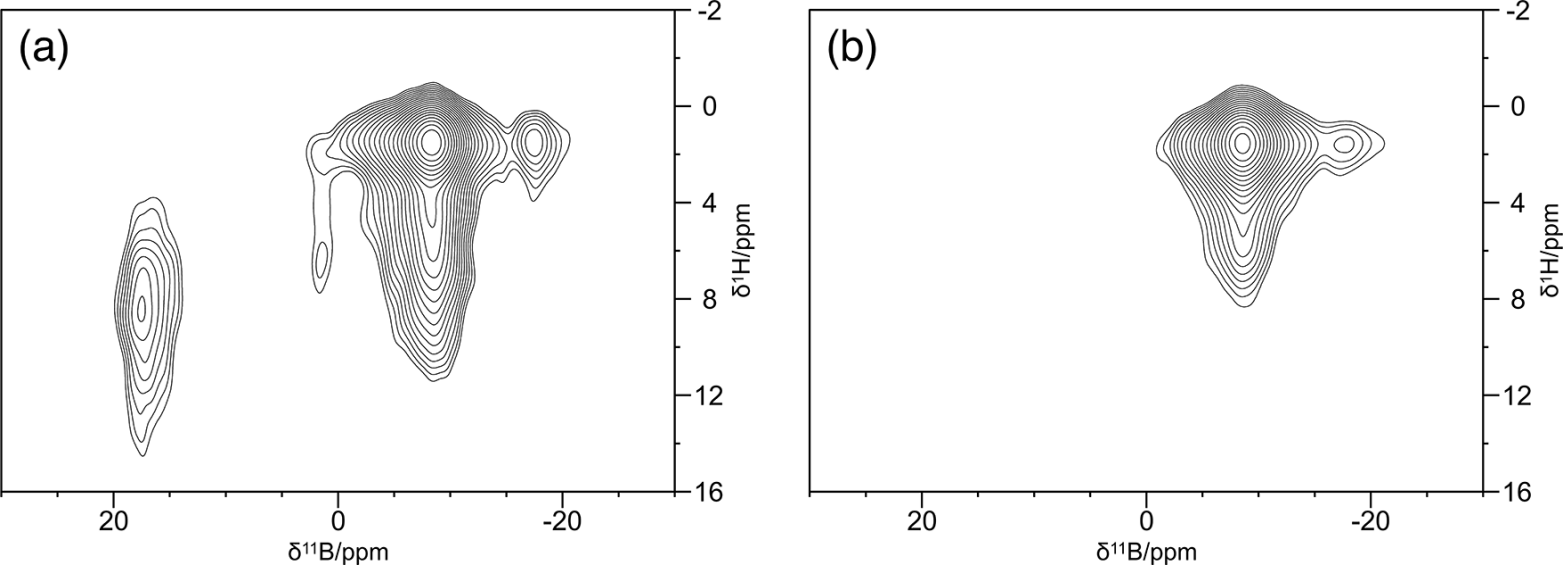
2D ^1^H–^11^B FSLG HETCOR spectra of [AB@**Be_BDC_NH**_**2**_], with a contact time of (a) 400 μs (long contact time) and (b) 100 μs (short contact time).

**Figure 13:**
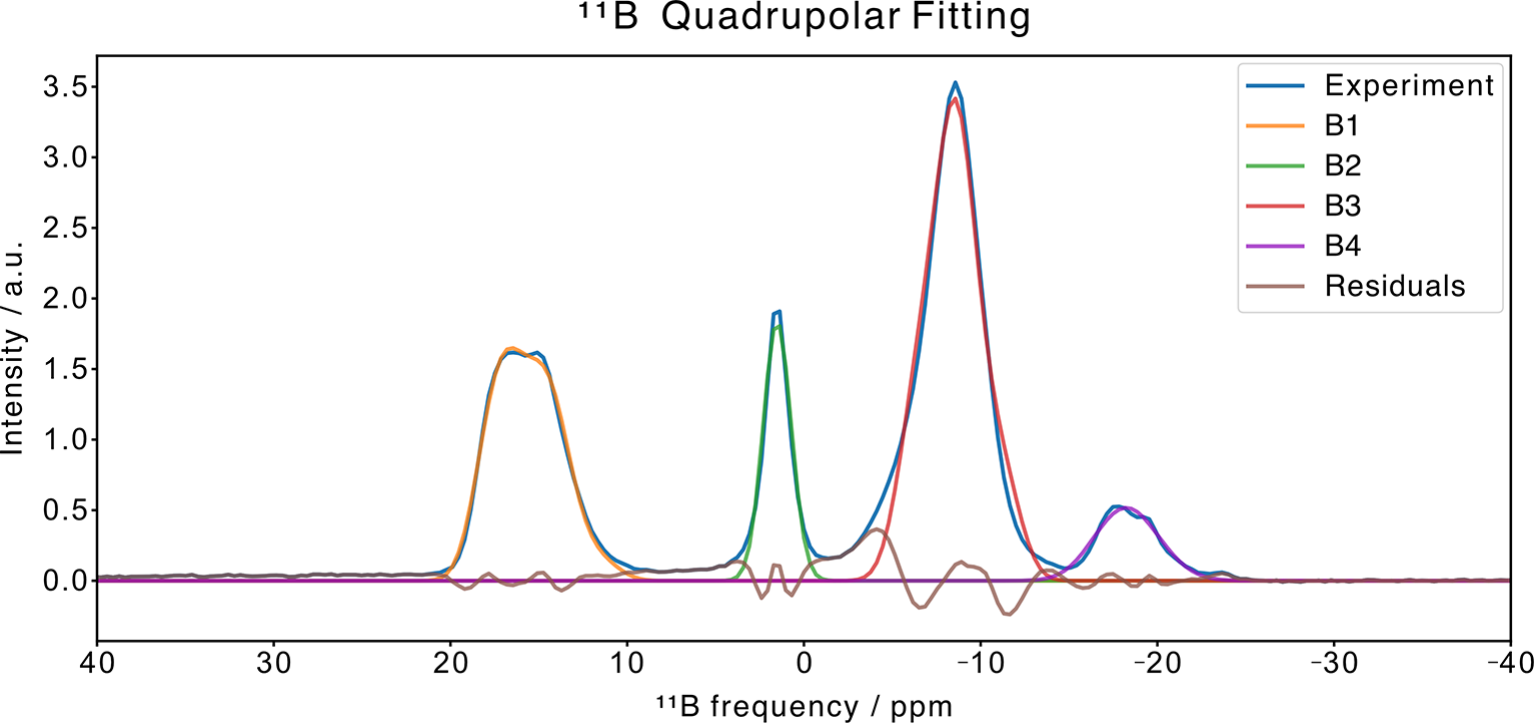
Quadrupolar fitting of 1D ^11^B Hahn-Echo decoupling MAS spectrum of [AB@**Be_BDC_NH**_**2**_] fitted with MrSimulator.^40^

**Table 2:** ^11^B Isotropic Chemical Shift (^iso^δ_B_), Nuclear Electric Quadrupolar Coupling Constant (*C*_Q_) and Asymmetry Parameter (η_Q_) Values Extrapolated from the Fitting of 1D ^11^B Hahn-Echo Decoupling MAS Spectrum of [AB@**Be_BDC_NH**_**2**_]

	^iso^δ_B_ (ppm)	*C*_Q_ (MHz)	η_Q_	Gaussian fwhm (Hz)
**B1**	19.1 ± 0.2	2.61 ± 0.04	0.3 ± 0.1	(5 ± 1) × 10^2^
**B2**	2 ± 2	1.1 ± 1.9		(4 ± 8) × 10^2^
**B3**	–5.42 ± 0.06	2.2 ± 0.1	1.0 ± 0.2	(49 ± 2) × 10^1^
**B4**	–16 ± 3	2.1 ± 0.8		(7 ± 7) × 10^2^

**Scheme 2:**
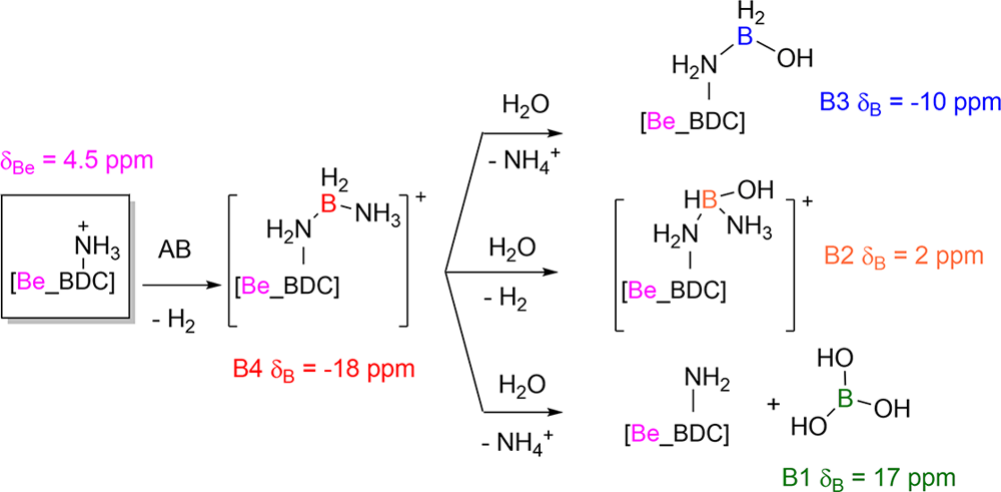
Proposed reaction scheme between AB and the dangling amino group on the MOF linker, based on the ^11^B NMR MAS peak assignment.

The {^1^H}-^15^N CP-MAS NMR spectrum of [AB@**Be_BDC_NH**_**2**_] recorded at natural isotopic abundance (Figure 14) shows a sharp signal at δ_N_ = 85 ppm, along with a broad distribution of signals in the range 55 ppm ≤ δ_N_ ≤ 80 ppm. The sharp signal at δ_N_ = 85 ppm can be assigned to the neutral amino group of BDC-NH_2_.^51^ The broad signal in the 50 ppm ≤ δ_N_ ≤ 75 ppm range indicates a distribution of nitrogen environments that could be assigned to the different BN-containing molecules present in the MOF. This is in agreement with the value of δ_N_ = 46 ppm found for the [Zr]-O-BH_2_–NH_3_ dangling group previously published.^47^

**Figure 14:**
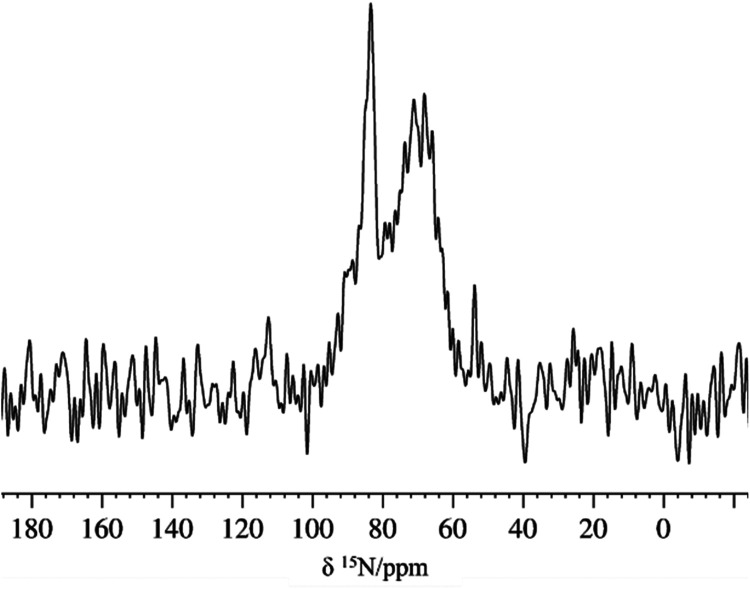
Natural abundance 1D {^1^H}-^15^N CP-MAS spectrum of [AB@**Be_BDC_NH**_**2**_] with a long contact time (600 μs).

Finally, the amount of boron loaded in the MOF was quantified through signal integration in the ^11^B Hahn-Echo with ^1^H decoupling spectra (Table 3). The boron signal integrals of [AB@**Be_BDC_NH**_**2**_] were compared with the integral measured in a pure solid AB batch taken as reference standard and acquired under the same experimental conditions, with a sufficiently long recycle delay to ensure complete relaxation. The background signals were subtracted from each spectrum. Based on the relative integrals and the known sample mass (0.0068 g), the gravimetric content (wt %) of boron species corresponding to each individual signal was calculated. The relative error of the integration was estimated by evaluating the integral of the noise in spectral regions analogous to that used to integrate the signal but containing only noise. The resulting gravimetric hydride contents were 6.7 ± 0.2 wt % (B1), 2.17 ± 0.07 wt % (B2), 5.8 ± 0.2 wt % (B3), and 0.97 ± 0.03 wt % (B4). These values were then converted into the number of molecules per MOF formula unit, assuming the latter as [(Be_4_O)­(BDC-NH_2_)_2.5_(OAc)] (FW = 558.97 g/mol). Thus, if the collective B1–B4 amount is considered, the minimal formula can be written as [(Be_4_O)­(BDC-NH_2_)_2.5_(OAc)] × 2.1­(AB).

**Table 3:** Experimental Parameters Used to Quantify the Amount of Boron Hydrides Loaded in [AB@**Be_BDC_NH**_**2**_]­^a^

boron species	boron amount [wt %]	total amount [wt %]	# of molecules
**B1**	1.17 ± 0.04	6.7 ± 0.2	0.61 ± 0.02
**B2**	0.50 ± 0.02	2.17 ± 0.07	0.259 ± 0.008
**B3**	2.05 ± 0.06	5.8 ± 0.2	1.06 ± 0.03
**B4**	0.34 ± 0.01	0.97 ± 0.03	0.176 ± 0.006

a The relative integrals were obtained with respect to those of boron in pure AB.

## 4 Conclusions

Summarizing, **Be_BDC_NH**_**2**_, a beryllium-based metal–organic framework with a linker bearing a basic amino group has been successfully synthesized and comprehensively characterized. This MOF combines low molecular weight (beryllium is the second lightest metallic element in the periodic table), high surface area and remarkable thermal stability, making it an effective platform for both physical and chemical hydrogen storage. The reversible hydrogen physisorption observed at cryogenic temperatures confirms the suitability of **Be_BDC_NH**_**2**_ as an efficient sorbent, with an H_2_ isosteric heat of adsorption comparable to other beryllium MOFs of the literature. Chemical hydrogen storage achieved through impregnation with the BN-based inorganic hydride ammonia borane revealed significant pore filling boosted by an initial N–H···H–B dihydrogen bonding between ammonia borane and the dangling −NH_2_ group on the linker. The formation of diverse boron-containing species after H_2_ thermal release is demonstrated by ^11^B and ^15^N solid-state NMR. Importantly, this work represents the first example of a “hybrid” physical and chemical hydrogen storage within a single material, opening new horizons for the preparation of better-performant MOFs for hydrogen storage technologies. In our laboratories, we are currently turning our attention to the preparation of other lightweight MOFs also based on less toxic metals like aluminum or magnesium for the same application and a more viable practical use.

## Supplementary Material





## Notes

Additional characterization data of **Be_BDC_NH**_**2**_ (IR spectra, TG-MS profiles, sequential H_2_ adsorption/desorption cycles carried out at *T* = 87 K, ^1^H and ^13^C NMR assignment of 2-aminoterephtalic acid, AB decomposition products assignment in the related ^11^B NMR spectrum in solution, tables of NMR experimental parameters) (PDF)
Crystallographic details for **Be_BDC_NH**_**2**_ and Rietveld refinement graphical representation (CIF)
