# Supplementary material for: A Lightweight Beryllium Metal–Organic Framework for Combined Physical and Chemical Hydrogen Storage
Source: ACS Appl Energy Mater. 2025 Oct 31;8(22):16891–903. doi: 10.1021/acsaem.5c02864 (PMC12648466; doi:10.1021/acsaem.5c02864)
Supplement: Supplementary file 1 [file ae5c02864_si_001.pdf]

Supporting Information

A Lightweight Beryllium Metal-Organic Framework for Combined Physical and Chemical Hydrogen Storage

Giacomo Provinciali,[a] *Naomi Anna Consoli,*[b,c,d] *Martino Degli Innocenti,*[b,c,d] *Anna Moliterni,*[e] *Heryson Tresmann,*[f] *Rocco Caliandro,*[e] *Cinzia Giannini,*[e] *Rolando Pedicini,*[f] *Giuliano Giambastiani,*[c,g,a] *Giulia Tuci,*[a,g] *Moreno Lelli* [b,c,d] *and Andrea Rossin**[a,g]

[a] Istituto di Chimica dei Composti Organometallici (CNR-ICCOM), Via Madonna del Piano 10, Sesto Fiorentino (Firenze), 50019, Italy. E-mail: a.rossin@iccom.cnr.it

[b] Centre of Magnetic Resonance (CERM) Università di Firenze, Via Luigi Sacconi 6, Sesto Fiorentino (Firenze), 50019, Italy.

[c] Dipartimento di Chimica "Ugo Schiff", Università di Firenze, Via della Lastruccia 3-13, 50019 Sesto Fiorentino (Firenze), Italy.

[d] Consorzio Interuniversitario Risonanze Magnetiche Metallo Proteine (CIRMMP), Via Luigi Sacconi 6, Sesto Fiorentino (Firenze), 50019, Italy.

[e] Istituto di Cristallografia (CNR-IC), via Amendola 122/o 70125 Bari (Italy).

[f] CNR-ITAE, Institute for Advanced Energy Technologies, Via S. Lucia sopra Contesse 5, 98126 Messina, Italy.

[g] Consorzio Interuniversitario Nazionale per la Scienza e Tecnologia dei Materiali (INSTM), Via G. Giusti, 9 50121 Firenze, Italy.

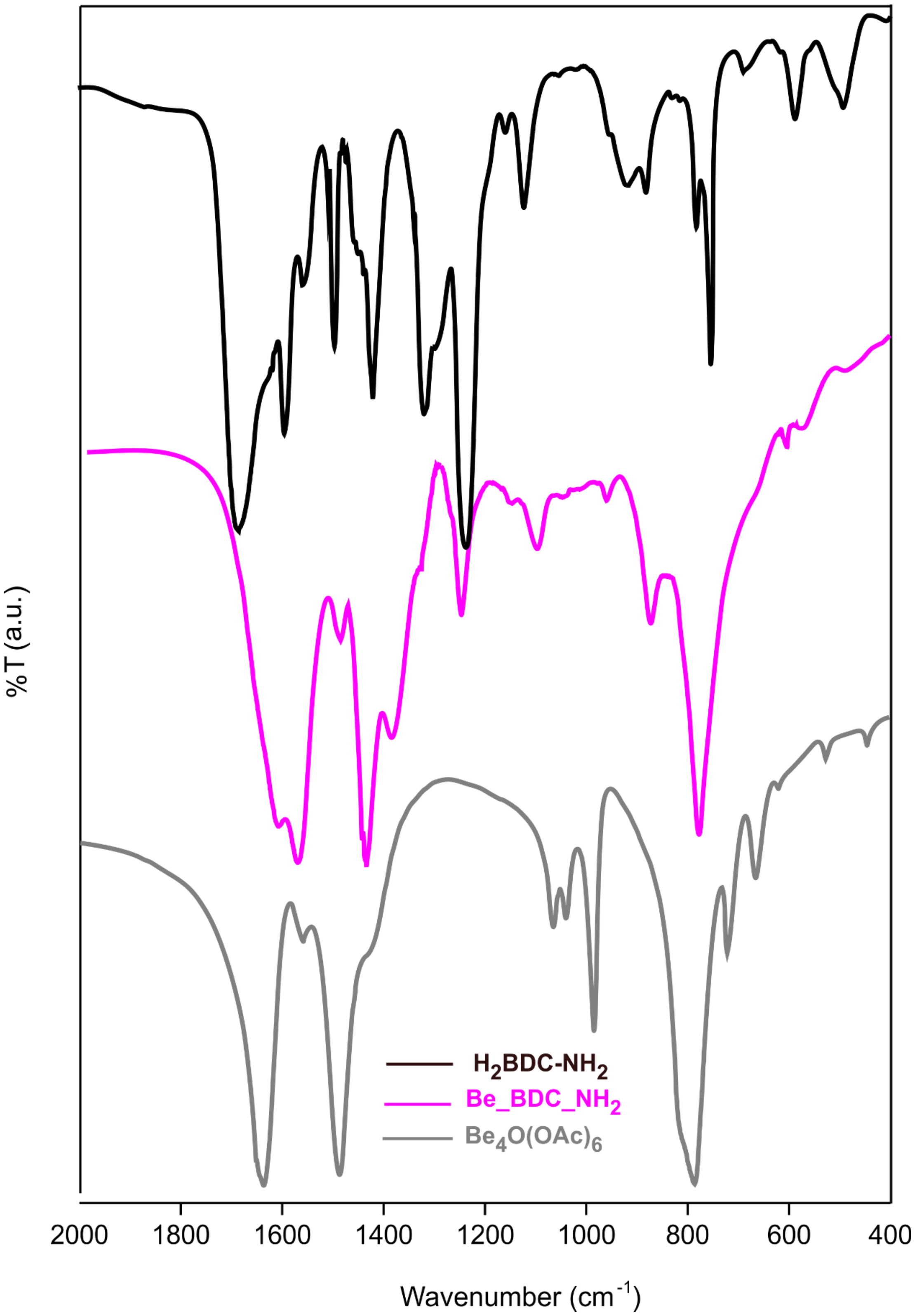

Figure S1. Infrared spectra (KBr, T = 298 K, 2000-400 cm^{-1}) of **Be_BDC_NH2** and its constitutive parts H_2BDC-NH_2 and beryllium acetate basic at comparison.

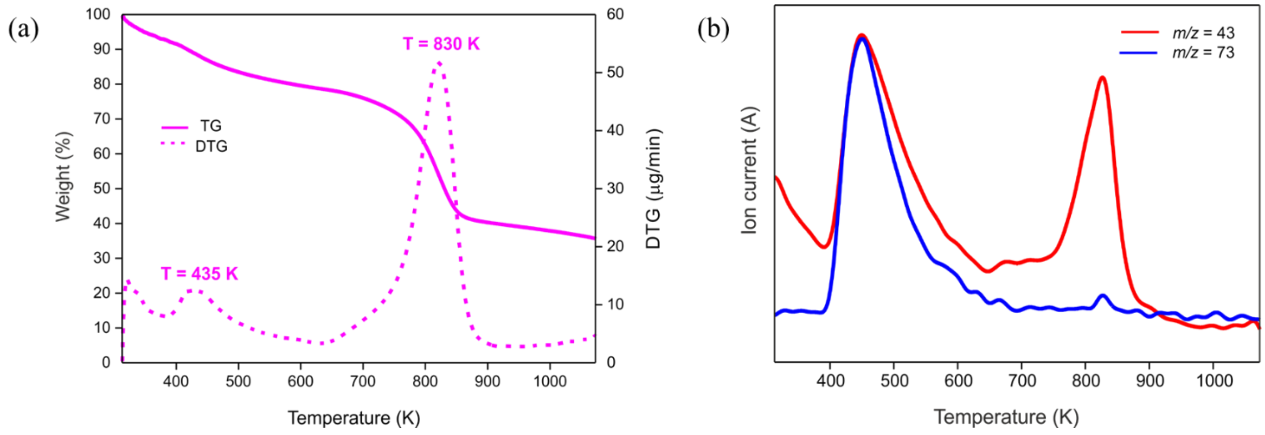

Figure S2. TG-DTG traces (a) and related mass spectrometry peaks (b) of **Be_BDC_NH$_2$**.

Tables of NMR Experimental Parameters

Table S1. Acquisition parameters for ^{1}H-^{13}C Cross-Polarization MAS (CP-MAS) and ^{1}H-^{13}C FSLG HETCOR at 16.4 T (701 MHz) spectrometer for **Be_BDC_NH₂**, 3.2 mm probe.

**Experimental Parameters**	**^{1}H**	**^{13}C**		
calibrated π/2 pulse (μs)	2.00	3.75		
	**CP-MAS**	**HETCOR**		
Acquired Nuclei	^{13}C	^{1}H		^{13}C
MAS Frequency (kHz)	20.0	20.0		
Number of scans	10240	512		
Recycle delay (s)	3.0	2.5		
Spectral width (kHz)	74.63	42.37		74.63
Acquisition time (ms)	20.6	1.2*	0.8**	13.7
^{1}H CP sequence	ramp 70% to 100%	ramp 70% to 100%		-
^{1}H CP power (100%) (kHz)	88.9	88.9		-
^{13}C CP sequence	constant power	-		constant power
^{13}C CP power (kHz)	77.0	-		77.0
^{1}H Decoupling sequence	SPINAL-64[a]	SPINAL-64[a]		-
^{1}H Decoupling power (kHz)	80.0	80.0		-
^{1}H homonuclear Power (FSLG)[b] (kHz)	-	80.0		-
Real acquired points	3072	100*	66**	2048
Window functions	Exponential (LB: 150 Hz)	Square Sine Bell (SSB: 2)		Exponential (LB: 400 Hz)
Real processed points	262144	1024		2048

* CP Contact time = 50 μs. ** CP Contact time = 1000 μs.

[a] Fung, B. M.; Khitrin, A. K.; Ermolaev, K. *J. Magn. Res.* **2000**, *142*, 97–101. [b] Lee, M.; Goldburg, W.I. *Phys. Rev.* **1965**, *140*,1261-1271.

Table S2. Acquisition parameters for ^{13}C 1D Hahn-echo with ^{1}H decoupling at 16.4 T (701 MHz) spectrometer for **Be_BDC_NH$_2$**, 3.2 mm probe.

**Hahn Echo Experimental Parameters**	**^{1}H**	**^{13}C**
calibrated π/2 pulse (μs)	2.00	3.75
Acquired Nucleus		^{13}C
MAS Frequency (kHz)		20.0
Number of scans		8960
Recycle delay (s)		20.0
Spectral width (kHz)		86.21
Acquisition time (ms)		18.0
^{1}H Decoupling sequence	SPINAL-64[a]	-
^{1}H Decoupling power (kHz)	80.0	-
Real acquired points		3072
Window functions		Exponential (LB: 150 Hz)
Real processed points		262144

[a] Fung, B. M.; Khitrin, A. K.; Ermolaev, K. *J. Magn. Res.* **2000**, *142*, 97–101.

Table S3. Acquisition parameters for ^{9}Be 1D Hahn-echo with ^{1}H decoupling and ^{1}H-^{9}Be FSLG HETCOR at 16.4 T (701 MHz) spectrometer for **Be_BDC_NH$_2$**.

<table>
<tr><th>Experimental Parameters</th><th>^{1}H</th><th colspan="3">^{9}Be</th></tr>
<tr><td>Calibrated π/2 pulse (μs)</td><td>2.00</td><td colspan="3">6.00</td></tr>
<tr><td></td><td>**Hahn Echo**</td><td colspan="3">**HETCOR**</td></tr>
<tr><td>Acquired Nuclei</td><td>^{9}Be</td><td colspan="2">^{1}H</td><td>^{9}Be</td></tr>
<tr><td>MAS Frequency (kHz)</td><td>20.0</td><td colspan="3">20.0</td></tr>
<tr><td>Number of scans</td><td>80</td><td colspan="3">256</td></tr>
<tr><td>Recycle delay (s)</td><td>10.0</td><td colspan="3">2.5</td></tr>
<tr><td>Spectral width (kHz)</td><td>39.68</td><td colspan="2">23.84</td><td>30.12</td></tr>
<tr><td>Acquisition time (ms)</td><td>51.61</td><td colspan="2">1.5</td><td>34.0</td></tr>
<tr><td>^{1}H CP sequence</td><td>-</td><td colspan="2">ramp 70 to 100%</td><td>-</td></tr>
<tr><td>^{1}H CP power (100%) (kHz)</td><td>-</td><td colspan="2">108.9</td><td>-</td></tr>
<tr><td>^{11}B CP sequence</td><td>-</td><td colspan="2">-</td><td>constant power</td></tr>
<tr><td>^{11}B CP power (kHz)</td><td>-</td><td colspan="2">-</td><td>34.7</td></tr>
<tr><td>^{1}H Decoupling sequence</td><td>-</td><td colspan="2">SPINAL-64[a]</td><td></td></tr>
<tr><td>^{1}H Decoupling power (kHz)</td><td>-</td><td colspan="2">70.0</td><td>-</td></tr>
<tr><td>^{1}H homonuclear Power (FSLG)[b] (kHz)</td><td>-</td><td colspan="2">80.0</td><td>-</td></tr>
<tr><td>Real acquired points</td><td>4096</td><td>72*</td><td>100**</td><td>2048</td></tr>
<tr><td>Window functions</td><td>Exponential (LB: 5 Hz)</td><td colspan="2">Square Sine Bell (SSB: 2)</td><td>Exponential (LB: 80 Hz)</td></tr>
<tr><td>Real processed points</td><td>8192</td><td colspan="2">1024</td><td>2048</td></tr>
</table>

* CP Contact time = 50 μs. ** CP Contact time = 1500 μs.

[a] Fung, B. M.; Khitrin, A. K.; Ermolaev, K. *J. Magn. Res.* **2000**, *142*, 97–101. [b] Lee, M; Goldburg, W. I., *Phys. Rev.* **1965**, *140*, 1261-1271.

Table S4. Acquisition parameters for ^{1}H-^{13}C Cross-Polarization MAS (CP-MAS) and ^{1}H-^{13}C FSLG HETCOR at 20.0 T (850 MHz) spectrometer for [AB@**Be_BDC_NH₂**], 3.2 mm probe.

<table>
<tr><th>Experimental Parameters</th><th>^{1}H</th><th colspan="8">^{13}C</th></tr>
<tr><td>calibrated π/2 pulse (μs)</td><td>2.40</td><td colspan="8">3.50</td></tr>
<tr><td></td><td>CP-MAS</td><td colspan="8">HETCOR</td></tr>
<tr><td>Acquired Nuclei</td><td>^{13}C</td><td colspan="4">^{1}H</td><td colspan="4">^{13}C</td></tr>
<tr><td>MAS Frequency (kHz)</td><td>20.0</td><td colspan="3">14.0$^{\alpha}$</td><td colspan="2">20.0$^{\beta}$</td><td colspan="3">12.6$^{\gamma}$</td></tr>
<tr><td>Number of scans</td><td>20480</td><td colspan="3">512$^{\alpha}$</td><td colspan="2">2048$^{\beta}$</td><td colspan="3">1120$^{\gamma}$</td></tr>
<tr><td>Recycle delay (s)</td><td>2.5</td><td colspan="8">2.5</td></tr>
<tr><td>Spectral width (kHz)</td><td>150.00</td><td colspan="2">45.55$^{\alpha,\beta}$</td><td colspan="2">52.96$^{\gamma}$</td><td colspan="2">119.05$^{\alpha,\beta}$</td><td colspan="2">150.00$^{\gamma}$</td></tr>
<tr><td>Acquisition time (ms)</td><td>6.8</td><td colspan="2">0.6$^{\alpha,\beta}$</td><td colspan="2">0.8$^{\gamma}$</td><td colspan="2">10.8$^{\alpha,\beta}$</td><td colspan="2">13.7$^{\gamma}$</td></tr>
<tr><td>^{1}H CP sequence</td><td>ramp 70% to 100%</td><td colspan="4">ramp 70% to 100%</td><td colspan="4">-</td></tr>
<tr><td>^{1}H CP power (100%) (kHz)</td><td>83.7</td><td>72.5$^{\alpha}$</td><td colspan="2">83.7$^{\beta}$</td><td>69.48$^{\gamma}$</td><td colspan="4">-</td></tr>
<tr><td>^{13}C CP sequence</td><td>constant power</td><td colspan="4">-</td><td colspan="4">constant power</td></tr>
<tr><td>^{13}C CP power (kHz)</td><td>47.5</td><td colspan="4">-</td><td>34.3$^{\alpha}$</td><td colspan="2">56.0$^{\beta}$</td><td>54.3$^{\gamma}$</td></tr>
<tr><td>^{1}H Decoupling sequence</td><td>SPINAL-64^{a}</td><td colspan="4">SPINAL-64^{a}</td><td colspan="4">-</td></tr>
<tr><td>^{1}H Decoupling power (kHz)</td><td>80.0</td><td colspan="4">80.0</td><td colspan="4">-</td></tr>
<tr><td>^{1}H homonuclear Power (FSLG)^{b} (kHz)</td><td>-</td><td colspan="2">86.0$^{\alpha,\beta}$</td><td colspan="2">100.0$^{\gamma}$</td><td colspan="4">-</td></tr>
<tr><td>Real acquired points</td><td>2048</td><td>50$^{\alpha}$</td><td colspan="2">56$^{\beta}$</td><td>80$^{\gamma}$</td><td colspan="2">2560$^{\alpha,\beta}$</td><td colspan="2">4096$^{\gamma}$</td></tr>
<tr><td>Window functions</td><td>Exponential
(LB: 150 Hz)</td><td colspan="4">Square Sine Bell
(SSB: 2)</td><td colspan="4">Exponential
(LB: 400 Hz)</td></tr>
<tr><td>Real processed points</td><td>131072</td><td colspan="4">1024</td><td colspan="4">4096</td></tr>
</table>

$^{\alpha}$ CP Contact time = 100 μs. $^{\beta}$ CP Contact time = 1000 μs. $^{\gamma}$ CP Contact time = 5000 μs.

^{a} Fung, B. M.; Khitrin, A. K.; Ermolaev, K. *J. Magn. Res.* **2000**, *142*, 97–101. ^{b} Lee, M.; Goldburg, W.I. *Phys. Rev.* **1965**, *140*,1261-1271.

Table S5. Acquisition parameters for ^{13}C 1D Hahn-echo with ^{1}H decoupling at 20.0 T (850 MHz) spectrometer for [AB@**Be_BDC_NH$_2$**], 3.2 mm probe.

**Hahn Echo Experimental Parameters**	**^{1}H**	**^{13}C**
calibrated π/2 pulse (μs)	2.40	3.50
Acquired Nucleus		^{13}C
MAS Frequency (kHz)		20.0
Number of scans		13056
Recycle delay (s)		12.0
Spectral width (kHz)		133.93
Acquisition time (ms)		15.3
^{1}H Decoupling sequence	SPINAL-64[a]	-
^{1}H Decoupling power (kHz)	79.8	-
Real acquired points		4096
Window functions		Exponential (LB: 150 Hz)
Real processed points		65536

[a] Fung, B. M.; Khitrin, A. K.; Ermolaev, K. *J. Magn. Res.* **2000**, *142*, 97–101.

Table S6. Acquisition parameters for ^{1}H-^{15}N Cross-Polarization MAS at 20.0 T (850 MHz) spectrometer for [AB@**Be_BDC_NH$_2$**], 3.2 mm probe.

**CP Experimental Parameters**	**^{1}H**	**^{15}N**
Calibrated π/2 pulse (μs)	2.00	7.40
Acquired Nuclei		^{15}N
MAS Frequency (kHz)		14.0
Number of scans		204800
Recycle delay (s)		2.5
Spectral width (kHz)		50.00
Acquisition time (ms)		10.2
^{1}H CP sequence	ramp 70% to 100%	
^{1}H CP power (100%) (kHz)	88.9	
^{15}N CP sequence		constant power
^{15}N CP power (kHz)		22.6
^{1}H Decoupling sequence	SPINAL-64[a]	
^{1}H Decoupling power (kHz)	70.0	
Real acquired points		2048
Window functions		Exponential (LB: 100 Hz)
Real processed points		32768

[a] Fung, B. M.; Khitrin, A. K.; Ermolaev, K. *J. Magn. Res.* **2000**, *142*, 97–101.

Table S7. Acquisition parameters for ^{11}B 1D Hahn-echo with ^{1}H decoupling for ^{11}B quantification and ^{1}H-^{11}B FSLG HETCOR at 16.4 T (701 MHz) spectrometer for [AB@**Be_BDC_NH₂**], 3.2 mm probe.

**Experimental Parameters**	**^{1}H**	**^{11}B**	
Calibrated π/2 pulse (μs)	2.00	3.20	
	**Hahn Echo**	**HETCOR**	
Acquired Nuclei	^{11}B	^{1}H	^{11}B
MAS Frequency (kHz)	20.0	20.0	
Number of scans	5856	128	
Recycle delay (s)	10.0	2.5	
Spectral width (kHz)	34.246	47.69	74.63
Acquisition time (ms)	15.0	0.8	13.7
^{1}H CP sequence	-	ramp 70 to 100%	-
^{1}H CP power (100%) (kHz)	-	92.0	-
^{11}B CP sequence	-	-	constant power
^{11}B CP power (kHz)	-	-	47.8
^{1}H Decoupling sequence	ttpm-15	SPINAL-64[a]	
^{1}H Decoupling power (kHz)	111.2	70.0	-
^{1}H homonuclear Power (FSLG)[b] (kHz)	-	90.0	-
Real acquired points	1024	80	2048
Window functions	Exponential (LB: 5 Hz)	Exponential (LB: 10 Hz)	Exponential (LB: 10 Hz)
Real processed points	65536	2048	4096

[a] Fung, B. M.; Khitrin, A. K.; Ermolaev, K. *J. Magn. Res.* **2000**, *142*, 97–101. [b] Lee, M; Goldburg, W. I., *Phys. Rev.* **1965**, *140*, 1261-1271.

Crystal Structure Determination of $Be\_BDC\_NH_2$ from X-ray Powder Diffraction Data

Indexing. The unit cell parameters was determined by *N-TREOR09*,[1] the default indexing program coded in *EXPO*,[2] using the information on 21 experimental diffraction peaks lying in the 6°-33° 2θ range. The most plausible cell found by *N-TREOR09* was cubic [cell parameters $a = b = c =$ 23.987(4) Å, cell volume $V = 13801(4)$ Å^3], characterized by a M_{20} de Wolff figure of merit[3] equal to 39 and zero unindexed lines, two values supporting the reliability of the cubic cell. The cell axis of **$Be\_BDC\_NH_2$** is slightly longer than that of MOF-5(Be),[4] [a = 23.72(2) Å], as expected due to the slightly larger number of atoms in the $BDC\text{-}NH_2^{2-}$ linker compared with BDC^{2-}. The cell parameter suggested by *N-TREOR09* will be further refined by Rietveld method on the last step of the structure determination process.

Space group determination. This step was carried out using the prior information on the unit cell parameters and on the atomic content by performing: (a) the integrated intensities extraction in $Pm\bar{3}m$ (*i.e.*, the space group with the largest Laue symmetry compatible with the geometry of the cubic unit cell and no extinction conditions); (b) a statistical analysis on the suitably weighted integrated intensities, to find the systematic absences and calculate a probability value for each extinction symbol compatible with the cubic crystal system.[5-6] *EXPO* correctly identified the extinction symbol $F - - -$ as the most plausible, and one of the five space groups referring to it ($Fm\bar{3}m$) was graphically selected to continue the *ab-initio* structure solution pathway.

Full pattern decomposition. The extraction of the integrated intensities of reflections was performed applying the Le Bail algorithm.[7]

Structure determination. The limited quality of the diffraction data (*e.g.*, peaks broadness, preferred orientation effects, low signal to noise ratio for 2θ > 50°) prevented the structure

determination through Direct Methods (the default approach of *EXPO*)[8] working in the reciprocal space. The availability of preliminary information on the expected geometry of the crystal structure and the similarity with MOF-5(Be)[4] allowed to successfully solve the structure in direct space through the Simulated Annealing (SA) approach implemented in *EXPO*,[9] exploiting the knowledge of unit cell parameters and space group and using two starting molecular fragments: the structural model of MOF-5(Be) and a trial amino (-NH_2) group randomly oriented in the cubic unit cell. Ten independent SA runs were carried out; the corresponding ten final candidate structure models were ranked according to increasing values of the R_{wp} default cost function: the weighted-profile reliability factor, the weighted sum of the squared differences between observed and calculated profile.[10] During each SA run, the MOF-5(Be) structural model was kept fixed, leaving only the position and the orientation of the center of mass of the NH_2 group (external Degrees of Freedom, DOFs) to be varied by *EXPO* to find the best configuration corresponding to the minimum of the cost-function hypersurface. Indeed, the DOFs total number for **Be_BDC_NH2** was six (only external DOFs), describing the position and the orientation of the center of mass of the NH_2 group. In addition, during each SA run a restraint on the C−N bond distance (1.377 Å) was applied, fixing it to the average value retrieved from the Cambridge Structural Database (CSD)[11] *via* the *ConQuest* software,[12]searching for structural models containing 2-aminoterephtalic acid. During the SA runs a correction for preferred orientation effects [plane (100)] was applied *via* the March-Dollase formula.[13] At the end of the SA procedure, the structure model with the lowest R_{wp} cost function was graphically selected for the next final step (Rietveld refinement).

Rietveld refinement. The selected best SA model of **Be_BDC_NH2** was refined through the Rietveld method, optimizing profile and structural parameters and applying restraints on bond distances. During the Rietveld refinement, (i) the symmetry-induced disorder of the NH_2 group on the four C positions (three of them symmetry equivalent) of the terephthalate ring (Figure 4b) was

taken into account by assigning to each atom in the NH_2 group a site occupancy factor (*SOF*, Table S9) equal to 0.25; (ii) the preferred orientation effects [plane (100)] were corrected *via* the March-Dollase formula.[13] Figure S3 shows the final Rietveld outcome characterized by R_p and R_{wp} agreement factors equal to 6.0% and 10.7%, respectively (Table S8).

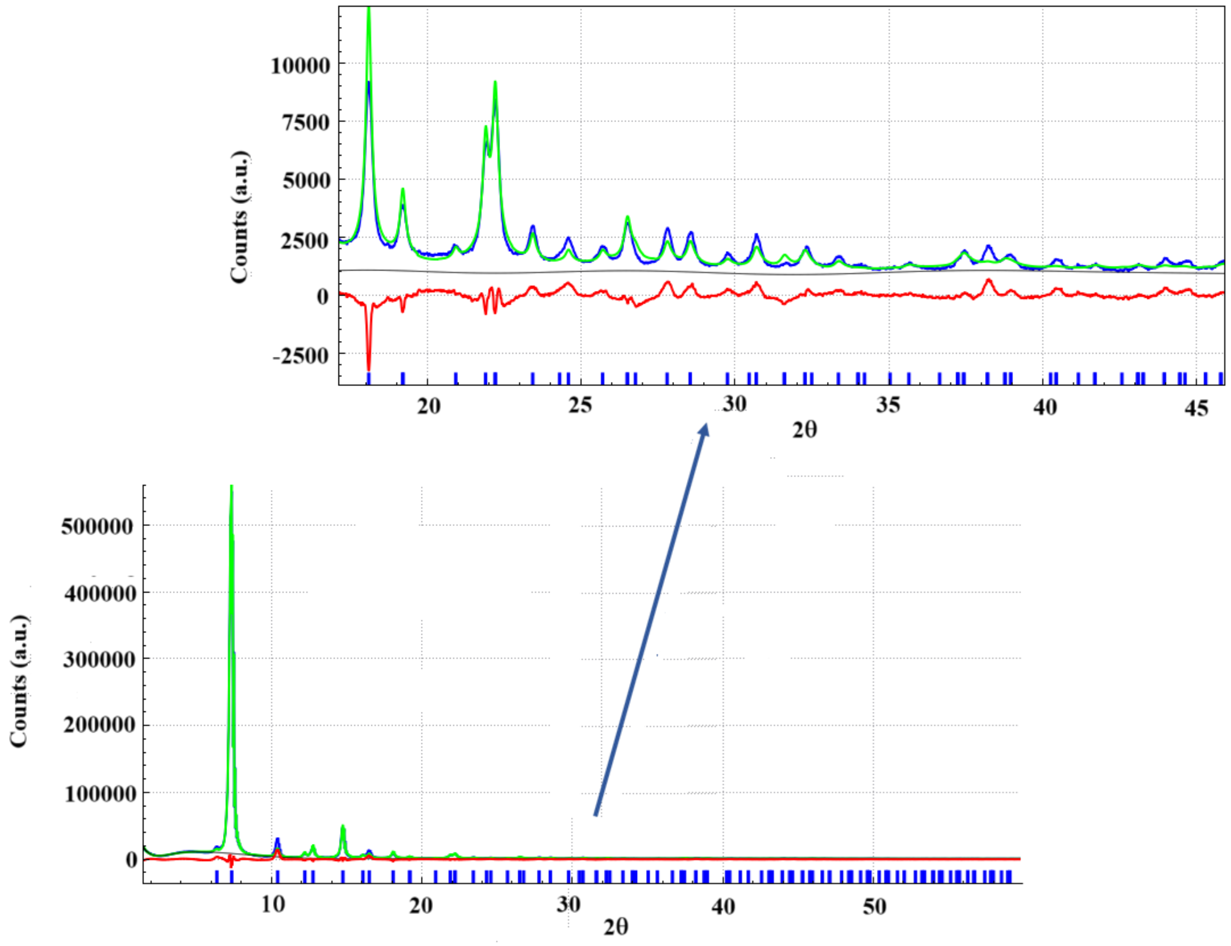

Figure S3. Final Rietveld refinement results of **Be_BDC_NH_2** showing the calculated (green), observed (blue), difference (red) pattern and the calculated 2θ-position of reflections (blue vertical bars at the bottom). A magnified view of an inner portion of the main plot is also shown.

Table S8. Crystal data, data collection and structure refinement details of **Be_BDC_NH$_2$**

Crystal data	
Chemical formula	$C_{24}H_6Be_4N_3O_{13}$
M_r	580.37
Crystal system, space group	Cubic, $Fm\bar{3}m$
Temperature (K)	293
a (Å)	24.004 (4)
V (Å^3)	13830 (4)
Z	8
Radiation type	Cu $K\alpha$
Data collection	
Diffractometer	Panalytical X'PERT PRO
Specimen mounting	Spinning circular sample holder (flat stage)
Data collection mode	Reflection
Scan method	θ : 2θ
2θ values (°)	$2\theta_{min}$ = 1.5; $2\theta_{max}$ = 90.00; $2\theta_{step}$ = 0.02
Refinement	
R factors	R_p = 0.060, R_{wp} = 0.107, R_{exp} = 0.012, R_{Bragg} = 0.109
No. data points	2925
No. of parameters	36
H-atom treatment	H-atom parameters constrained
Computer programs	***Applications***
EXPO[2]	Indexing, Space group determination, Structure solution (Direct Space Methods), Rietveld refinement, Molecular graphics
Mercury[14]	Molecular graphics
CheckCIF (https://checkcif.iucr.org)	CIF validation
publCIF[15]	Preparation of material for publication

Table S9. Fractional atomic coordinates (*x*, *y*, *z*), isotropic (U_{iso}) displacement parameters (Å^2), site occupancy factors (*SOF*) and selected geometric parameters (Å, °) of **Be_BDC_NH₂**. Refer to Figure 4 in the main text for atom numbering.

	x	***y***	***z***	***U*iso**	***SOF***
Be1	0.7018	0.2018	0.2018	0.0127	
O1	0.7500	0.2500	0.2500	0.0380	
O2	0.6348	0.2186	0.2186	0.0380	
C1	0.6100	0.2500	0.2500	0.0380	
C2	0.5258	0.2131	0.2131	0.0380	
C3	0.5549	0.2500	0.2500	0.0380	
N1	0.5574	0.1763	0.1763	0.0380	0.25000
H1	0.5915	0.1754	0.1754	0.0456	0.25000
H2	0.5365	0.1553	0.1553	0.0456	0.25000

Geometric parameters

Bond lengths (Å)			
Be1—O2	1.7064 (3)	C2—C2^{iv}	1.2386 (2)
Be1—O2^{i}	1.7064 (3)	C2—C3	1.4342 (2)
Be1—O2^{ii}	1.7064 (3)	C2—N1	1.4615
O2—C1	1.2209 (2)	C3—C2^{iii}	1.4342 (2)
C1—O2^{iii}	1.2209 (2)	N1—H1	0.8191
C1—C3	1.3226 (2)	N1—H2	0.8717
Bond angles (°)			
O2—Be1—O2^{i}	112.93	C1—C3—C2^{iii}	119.15 (1)
O2—Be1—O2^{ii}	112.93	C2^{iv}—C2—C3	119.15 (1)
Be1—O2—C1	138.71 (1)	C2^{iv}—C2—N1	121.27
O2^{i}—Be1—O2^{ii}	112.93	C3—C2—N1	119.59
O2—C1—O2^{iii}	121.64	C2—C3—C2^{iii}	121.71
O2—C1—C3	119.18 (1)	C2—N1—H1	123.40
O2^{iii}—C1—C3	119.18 (1)	C2—N1—H2	113.60
C1—C3—C2	119.15 (1)	H1—N1—H2	123.00
Dihedral angles (°)			
O2^{i}—Be1—O2—C1	-115.17	C2^{iv}—C2—C3—C1	180.00
O2^{ii}—Be1—O2—C1	115.17	C2^{iv}—C2—C3—C2^{iii}	0.00
Be1—O2—C1—O2^{iii}	0.00	N1—C2—C3—C1	0.00
Be1—O2—C1—C3	-180.00	N1—C2—C3—C2^{iii}	180.00
C2^{iv}—C2—N1—H2	-0.02	C2^{iv}—C2—N1—H1	-180.00

Symmetry codes: (i) *y*+1/2, *z*, *x*-1/2; (ii) *z*+1/2, *x*-1/2, *y*; (iii) *x*, -*y*+1/2, -*z*+1/2; (iv) -*x*+1, *z*, *y*.

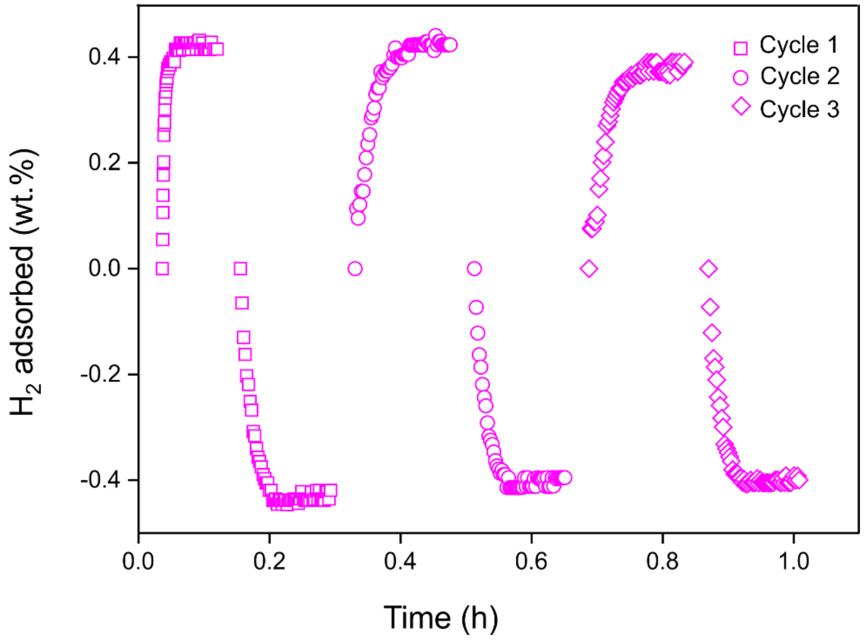

Figure S4. Sequential H_2 adsorption/desorption cycles carried out on **Be_BDC_NH₂** at p_{H2} = 10 bar, T = 87 K.

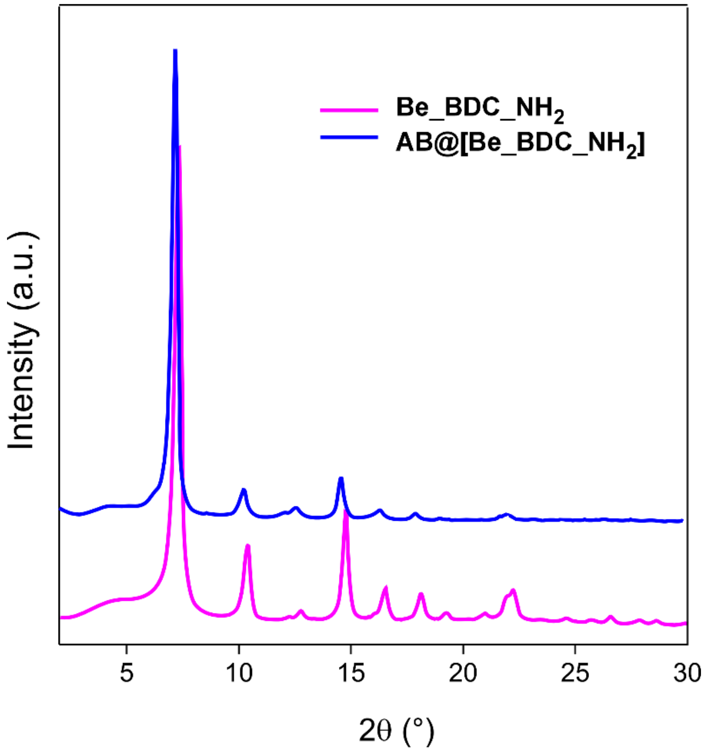

Figure S5. XRPD patterns (2-30° 2θ range) of **Be_BDC_NH$_2$** (magenta trace) and [AB@**Be_BDC_NH$_2$**] (blue trace) at comparison.

^{1}H and ^{13}C NMR assignment of 2-aminoterephtalic acid

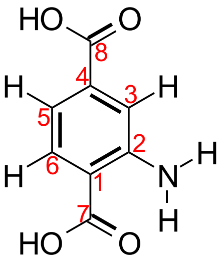

^{1}H NMR (600 MHz, DMSO-d_6, T = 298 K, ppm): δ 7.77 (d, $^{3}J_{HH}$ = 8.2 Hz, 1H, H^6); 7.38 (d, $^{4}J_{HH}$ = 1.5 Hz, 1H, H^3); 7.02 (dd, $^{3}J_{HH}$ = 8.2 Hz, $^{4}J_{HH}$ = 1.5 Hz, 1H, H^5). ^{13}C NMR (150 MHz, DMSO-d_6, T = 298 K, ppm): δ 169.6 (C^7), 167.7 (C^8), 151.7 (C^2), 135.6 (C^4), 131.9 (C^6), 118.0 (C^3), 115.1 (C^5), 112.9 (C^1).

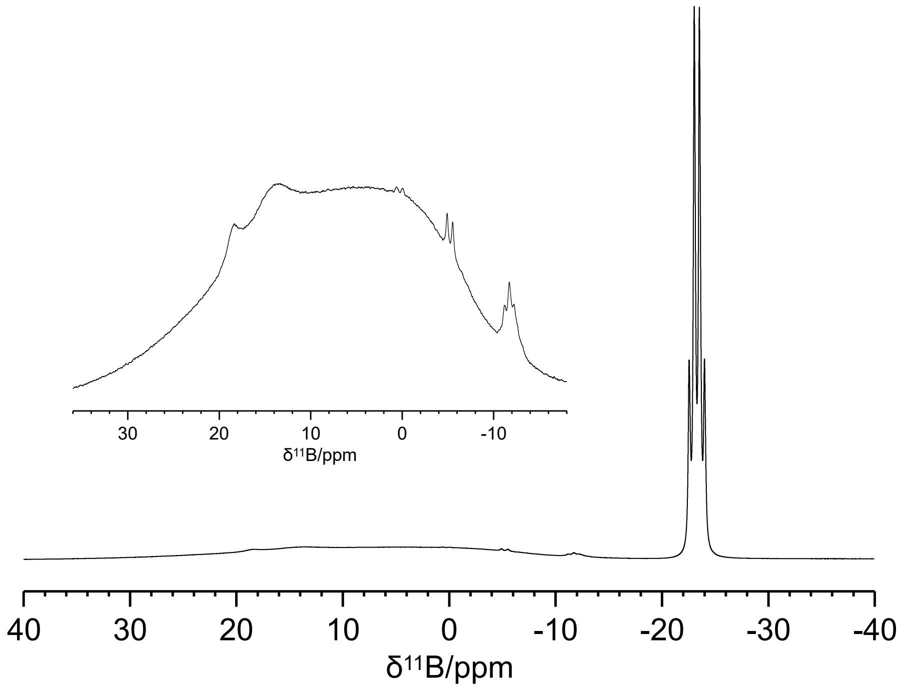

Figure S6. 1D ^{11}B solution state direct acquisition of AB in CD_3OD. Multiple spectra were acquired after five days, at 14.1 T (600 MHz of proton Larmor frequency) and at T = 298 K to check the stability of AB in methanol, and no appreciable degradation was observed. The present spectrum was acquired without ^{1}H decoupling, so that the number of ^{1}H atoms bound to ^{11}B can be determined from the resonance multiplicity. In addition to the quartet of AB at δ_B = –23 ppm, the resonance at δ_B = –10 ppm corresponds to a $[BH_2X_2]^+$ species (X = N, O) and those at δ_B = –5 and δ_B = 0 ppm are $[BHX_3]^+$ species. No directly bound hydrogens are observed for the signals at δ_B = 15 and δ_B = 19 ppm.

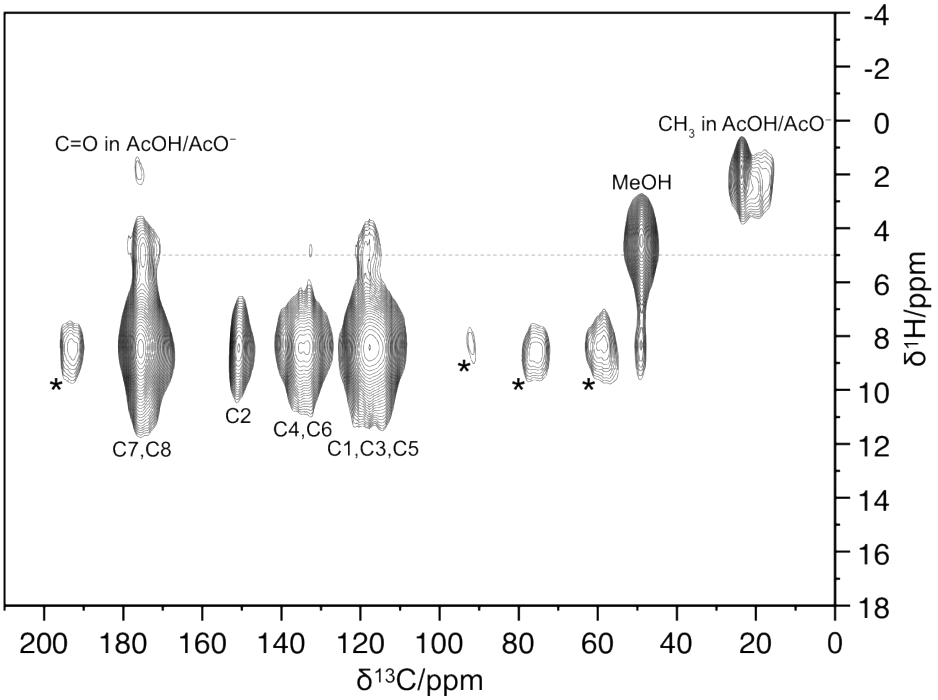

Figure S7. 2D ^{1}H-^{13}C FSLG HETCOR spectra of [AB@**Be_BDC_NH₂**] collected with a long contact time of 5000 μs. At this contact time it is possible to observe correlation between the acetic acid methyl protons and their carboxylate ^{13}C and the correlations between the $(BDC\text{-}NH_2)^{2-}$ carbons and protons at $\delta_H = 5$ ppm assigned to the ammonia part of AB. The dashed line evidences the difference from the newly detected ^{1}H resonance and the MeOH resonance. Asterisks denote spinning sidebands (MAS = 12600 Hz).

Solution ^{1}H NMR composition analysis on a digested MOF sample.

To confirm and quantify the presence of residual acetate in **Be_BDC_NH$_2$**, we prepared a fresh sample batch using the same synthetic procedure described in the main text. The sample was then washed and activated under vacuum using the same procedure followed before the collection of the N_2 adsorption isotherm (see the Experimental Section in the main text) to remove residual clathrate DMF solvent and acetic acid present in the pores. Then, *ca.* 6 mg of sample were digested by adding a few droplets of deuterated D_2SO_4 in D_2O and 0.5 mL of anhydrous DMSO-d_6. After complete solubilization, the sample was analyzed through quantitative solution ^{1}H NMR. The 1D NMR spectrum was acquired with a recycled delay of 8.0 s and an acquisition time of 1.18 s and 1024 scans (to ensure complete relaxation of the resonances) and confirmed the presence of residual acetate. Table S10 reports the integral values for the methyl acetate resonance (3H) and one resonance coming from the proton labelled as H^6 of the amino-terephthalate linker (see Figure 9 in the main text, 1H). From the relative integral of these two resonances, we established the MOF composition starting from a general stoichiometry [$Be_4O(BDC\text{-}NH_2)_x(OAc)_{6-2x}$] where x is determined from the relative integrals of the two resonances. The analysis reveals a composition corresponding to the formula [$Be_4O(BDC\text{-}NH_2)_{2.76}(OAc)_{0.48}$].

Table S10. Integral values used for linkers quantification.

	δ_H (ppm)	Integral (absolute units)	Relative integral
**Acetate (CH_3)**	1.70	$(8407 \pm 4) \cdot 10^{9}$	1
**BDC-NH_2 (H^6)**	7.93	$(1601 \pm 2) \cdot 10^{10}$	1.904

References

1. Altomare, A.; Campi, G.; Cuocci, C.; Eriksson, L.; Giacovazzo, C.; Moliterni, A.; Rizzi, R.; Werner, P.-E., Advances in Powder Diffraction Pattern Indexing: *N-TREOR09*. *J. Appl. Cryst.* **2009,** *42*, 768-775.
2. Altomare, A.; Cuocci, C.; Giacovazzo, C.; Moliterni, A.; Rizzi, R.; Corriero, N.; Falcicchio, A., *EXPO2013*: a Kit of Tools for Phasing Crystal Structures from Powder Data. *J. Appl. Cryst.* **2013,** *46*, 1231-1235.
3. De Wolff, P. M., A Simplified Criterion for the Reliability of a Powder Pattern Indexing. *J. Appl. Cryst.* **1968,** *1*, 108-113.
4. Hausdorf, S.; Baitalow, F.; Böhle, T.; Rafaja, D.; Mertens, F. O. R. L., Main-Group and Transition-Element IRMOF Homologues. *J. Am. Chem. Soc.* **2010,** *132*, 10978-10981.
5. Altomare, A.; Camalli, M.; Cuocci, C.; Giacovazzo, C.; Moliterni, A. G. G.; Rizzi, R., Advances in Space-Group Determination from Powder Diffraction Data. *J. Appl. Cryst.* **2007,** *40*, 743-748.
6. Altomare, A.; Caliandro, R.; Camalli, M.; Cuocci, C.; Da Silva, I.; Giacovazzo, C.; Moliterni, A. G. G.; Spagna, R., Space-Group Determination from Powder Diffraction Data: a Probabilistic Approach. *J. Appl. Cryst.* **2004,** *37*, 957-966.
7. Le Bail, A.; Duroy, H.; Fourquet, J. L., *Ab-Initio* Structure Determination of $LiSbWO_6$ by X-Ray Powder Diffraction. *Mat. Res. Bull.* **1988,** *23*, 447-452.
8. Giacovazzo, C., *Phasing in Crystallography - A Modern Perspective*. International Union of Crystallography/Oxford University Press: 2013. ISBN: 9780199686995
9. Kirkpatrick, S., Optimization by Simulated Annealing: Quantitative Studies. *J. Stat. Phys.* **1984,** *34*, 975-986.

10. Young, R. A., Introduction to the Rietveld Method. In *The Rietveld Method*, Young, R. A., Ed. Oxford University Press: New York, NY, USA: 1996; p 22. ISBN: 9780198559122

11. Groom, C. R.; Bruno, I. J.; Lightfoot, M. P.; Ward, S. C., The Cambridge Structural Database. *Acta Cryst.* **2016,** *B72*, 171-179.

12. Bruno, I. J.; Cole, J. C.; Edgington, P. R.; Kessler, M.; Macrae, C. F.; McCabe, P.; Pearson, J.; Taylor, R., New Software for Searching the Cambridge Structural Database and Visualizing Crystal Structures. *Acta Cryst.* **2002,** *B58*, 389-397.

13. Dollase, W. A., Correction of Intensities for Preferred Orientation in Powder Diffractometry: Application of the March Model. *J. Appl. Cryst.* **1986,** *19*, 267-272.

14. Macrae, C. F.; Sovago, I.; Cottrell, S. J.; Galek, P. T. A.; McCabe, P.; Pidcock, E.; Platings, M.; Shields, G. P.; Stevens, J. S.; Towler, M.; Wood, P. A., *Mercury 4.0*: from Visualization to Analysis, Design and Prediction. *J. Appl. Cryst.* **2020,** *53*, 226-235.

15. Westrip, S. P., *publCIF*: Software for Editing, Validating and Formatting Crystallographic Information Files. *J. Appl. Cryst.* **2010,** *43*, 920-925.
